# Long-read assembly of major histocompatibility complex and killer cell immunoglobulin-like receptor genome regions in cynomolgus macaque

**DOI:** 10.1186/s13062-022-00350-w

**Published:** 2022-11-29

**Authors:** Qingxiu Hu, Xiaoqi Huang, Yabin Jin, Rui Zhang, Aimin Zhao, Yiping Wang, Chenyun Zhou, Weixin Liu, Xunwei Liu, Chunhua Li, Guangyi Fan, Min Zhuo, Xiaoning Wang, Fei Ling, Wei Luo

**Affiliations:** 1 Guangdong Key Laboratory of Fermentation and Enzyme Engineering, School of Biology and Biological Engineering, South China University of Technology, Guangzhou, 510006 China; 2 The First People’s Hospital of Foshan, Sun Yat-sen University, Foshan, 528000 China; 3 BGI-Qingdao, BGI-Shenzhen, Qingdao, 266555 China; 4 National Clinic Center of Geriatric, The Chinese PLA General Hospital, Beijing, 100853 China

**Keywords:** Major histocompatibility complex, Killer cell immunoglobulin-like receptor, *Mafa-B*, Third-generation sequencing, Cynomolgus macaque

## Abstract

**Background:**

The major histocompatibility complex (MHC) and the killer cell immunoglobulin-like receptors (KIR) are key regulators of immune responses. The cynomolgus macaque, an Old World monkey species, can be applied as an important preclinical model for studying human diseases, including coronavirus disease 2019 (COVID-19). Several MHC-KIR combinations have been associated with either a poor or good prognosis. Therefore, macaques with a well-characterized immunogenetic profile may improve drug evaluation and speed up vaccine development. At present, a complete overview of the MHC and KIR haplotype organizations in cynomolgus macaques is lacking, and characterization by conventional techniques is hampered by the extensive expansion of the macaque MHC-B region that complicates the discrimination between genes and alleles.

**Methods:**

We assembled complete MHC and KIR genomic regions of cynomolgus macaque using third-generation long-read sequencing approach. We identified functional *Mafa-B* loci at the transcriptome level using locus-specific amplification in a cohort of 33 Vietnamese cynomolgus macaques.

**Results:**

This is the first physical mapping of complete *MHC* and *KIR* gene regions in a Vietnamese cynomolgus macaque. Furthermore, we identified four functional *Mafa-B* loci (*B2*, *B3*, *B5*, and *B6*) and showed that alleles of the Mafa-I*01, -B*056, -B*034, and -B*001 functional lineages, respectively, are highly frequent in the Vietnamese cynomolgus macaque population.

**Conclusion:**

The insights into the MHC and KIR haplotype organizations and the level of diversity may refine the selection of animals with specific genetic markers for future medical research.

**Supplementary Information:**

The online version contains supplementary material available at 10.1186/s13062-022-00350-w.

## Introduction

Macaque species, such as cynomolgus (*Macaca fascicularis, Mafa*) and rhesus macaques (*Macaca mulatta*, *Mamu*), show close phylogenetic proximity to humans, and they share a common ancestor with humans from approximately 25–33 million years ago [1]. Humans and macaques have a highly related immune system; therefore, macaques are frequently used as non-human primate models for preclinical testing [2]. The use of cynomolgus macaques in biomedical studies has increased due to the limited supply of Indian rhesus macaques after the export ban in 1978 [3]. Cynomolgus macaques are used to study various human diseases such as acquired immunodeficiency syndrome (AIDS) [4], tuberculosis [5], and coronavirus disease 2019 (COVID-19) [6], as well as transplantation [7] and vaccine development [8].

The major histocompatibility complex (MHC) in humans, referred to as the human leukocyte antigen (HLA), plays a crucial role in the innate and adaptive immune responses. HLA is divided into class I, II, and III regions. A single copy of *HLA-A, HLA-B and HLA-C* is present in the HLA region. The equivalents of *HLA-A and HLA-B* have been detected in macaques and are named *Mamu-A/Mafa-A, Mamu-B/Mafa-B*. To date, no orthologs of *HLA-C* have been identified in macaques. In macaques, the function of *HLA-G* has been replaced by *MHC-AG* [9]. The genomic organization of *HLA* and macaque *MHC* regions is comparable [10]. Although the number of classical class I *A* and *B* genes is increased in macaques due to multiple rounds of duplication [10]. In a rhesus macaque, the presence and physical order of two *Mamu-A* and nineteen *Mamu-B* loci have been recorded [11]. Transcriptome studies have shown that the content of *Mamu-A* and *Mamu-B* genes vary considerably within each haplotype, leading to different haplotype configurations [12–14]. A systematic nomenclature has been established for *MHC-A* genes [15, 16], and different *A* genes have been designated *A1* to *A8* [17, 18]. On a haplotype, a difference in *A* gene content and combinations has been demonstrated [18–21]. A haplotype can contain one or two *Mamu-A* genes with high transcription levels and up to five *Mamu-A* genes with low transcription levels [13, 14, 19]. More complex gene content and variable transcript levels have been documented for the *MHC-B* region of macaques [13, 14, 20, 21]. The *Mamu-B* haplotype contains one to six major transcribed and one to ten minor transcribed *Mamu-B* genes [13, 14]. As a result, it is not yet possible to assign different *B* alleles to a particular *B* gene on a haplotype, and therefore *B* alleles are named according to the order in which they are discovered on the chromosome [22]. In addition, specific *MHC-B* alleles have been associated with the progression of several diseases. In humans, HLA-B*35, HLA-B*58, HLA-B*27 and HLA-B*57 showed robust correlations with HIV. For example, HLA-B*35 and HLA-B*58 are associated with rapid disease progression [23]. HLA-B*57 and HLA-B*27 are associated with slower disease progression and lower viral loads [24]. In rhesus macaques, Mamu-B*008 and Mamu-B*017 are known as protective alleles; individuals carrying Mamu-B*08 or Mamu-B*017 exhibited lower viral load and slower disease progression after SIVmac251/SIVmac239 challenge [25, 26]. Interestingly, Mamu-B*08/Mamu-B*017 restricted SIV-derived epitopes share a significant overlap with the peptide binding profile of HLA-B*27/HLA-B*57 [27, 28]. Mamu-B*001 is known to be a protective allele for collagen-induced arthritis (CIA) [29]. Additionally, Mamu-B*001 and Mamu-B*017 are distributed at high frequencies [29, 30]. Macaques carrying these high-frequency alleles are helpful in studying immune protection against various diseases.

MHC class I molecules are the predominant ligands of the killer cell immunoglobulin-like receptor (*KIR*) family and specific *MHC-KIR* interactions may be associated with health and disease [31, 32]. The genes encoding MHC and KIR are highly polymorphic, reflected by allelic and copy number variation. *KIRs* are expressed on natural killer (NK) cells and a subset of T cells [33, 34] and may interact with *MHC* class I molecules to transduce either an inhibitory or activating signal [35]. *KIR* genes are located on the leukocyte receptor complex (LRC) on chromosome 19 [36]. The human *KIR* region has been thoroughly characterized [37, 38] and consists of 17 genes, including 15 expressed genes and 2 pseudogenes [39]. Four genes (*KIR3DL3*, *KIR3DP1*, *KIR2DL4*, and *KIR3DL2*) are present in all human *KIR* haplotypes and are referred to as “framework genes” [40]. In humans, *KIR* haplotypes are divided into two categories, namely group A and group B haplotypes. Group A haplotypes contain a fixed set of seven *KIR* genes, whereas group B haplotypes contain a more significant variability in the number of genes [41]. Significant similarities have been observed between macaques and their human counterparts [42]. The macaque *KIR3DL20* has been detected in all haplotypes and is considered to originate from a common progenitor gene *KIR3DL3* in humans [43]. The ortholog of human *KIR2DL4* has been termed *KIR2DL04* in macaques [44]. A significant difference between human and macaque *KIRs* is that the KIR lineage II family in macaques has undergone intensive duplication, whereas the expansion of *KIR* in humans mainly involves lineage III [45]. The KIR region has been studied in rhesus macaques at the genomic [46, 47] and transcriptomic [42, 48, 49]. Multiple studies have sequenced *KIR* complementary cDNA sequences and used segregation analysis to detect the gene content of each *KIR* haplotype, showing that different individuals and rhesus macaques of different populations possess diverse *KIR* gene content [42, 48–52]. The number of *KIR* genes expressed per animal varies from 4 to 17 in rhesus macaques and 3–13 in cynomolgus macaques [42, 48, 49]. Multiple mechanisms have been shown to drive high variability in the *KIR* gene system, as evidenced by chromosomal recombination, point mutations, alternative splicing, and stochastic expression [53–55]. Given the complexity of *KIR* genes, long-read sequencing methods are required to improve the quality and continuity of genome assemblies. The combination of Cas9 enrichment and Oxford Nanopore Technologies (ONT) sequencing methods achieved allele-level resolution, which allowed the phasing of six *KIR* haplotypes in three rhesus macaques [47]. At present, cynomolgus macaque *KIR* has only been thoroughly studied at the transcriptomic level [48, 56]. However, these transcriptome studies show an unparalleled rapid evolution of the *KIR* gene region in macaques.

The first human genomic *HLA* region was successfully sequenced and fully annotated in 1999 [57], whereas a 5.1 Mb genome sequence of the rhesus monkey *MHC* was constructed and published in 2004 [11]. In a cynomolgus macaque, a BAC contig containing the *MHC* region was sequenced using a short-read sequencing approach in 2007 [58]. However, the short reads and *MHC* class I gene duplications resulted in the poor characterization of this region in cynomolgus macaques. Previously, we sequenced the genomes of a cynomolgus macaque and a Chinese rhesus macaque using a whole-genome shotgun strategy on the Illumina HiSeq (2000) platform [59]. However, the quality of the *MHC* genome assembly was poor because of the limitations of the sequencing technology. Currently, genome assemblies benefit from third-generation sequencing platforms with high accuracy and long read length [60]. For instance, the human *MHC* region has been characterized in over 20,000 individuals of Han Chinese ancestry using deep sequencing [61]. One study sequenced the *KIR* region using single-molecule real-time sequencing (SMRT) and phased 16 human *KIR* haplotypes [37]. Another study designed 18 probes to capture the *KIR* region of 16 samples and successfully assembled human diploid *KIR* haplotypes using long-read sequencing. The assemblies covered 97% of the reference genome with 99.97% sequence identity [38]. A high-quality Chinese rhesus macaque reference genome (rheMacs) was built by combining long-read sequencing and multi-platform scaffolding approaches [62]. A new version of the Indian rhesus monkey reference genome (Mmul_10) was assembled using SMRT sequencing, with 66-fold sequencing coverage and 120-fold increase in sequence continuity, as well as high-resolution annotations of *MHC* and *KIR* regions [63]. Jayakumar et al. assembled a high-fidelity chromosome-scale cynomolgus monkey genome that was superior in continuity and accuracy [64]. The human HG002/NA24385 genome, which was characterized by highly accurate circular consensus sequencing (CCS) of long reads, performed better in assembly quality and genetic variant detection [65]. The continuous development of third-generation long-read sequencing technologies advances the characterization of complete *MHC* and *KIR* gene regions.

This study aimed to assemble complete *MHC* and *KIR* genomic regions of a cynomolgus macaque using third-generation long-read sequencing technology.

## Materials and methods

### Animals and cells

For long-read sequencing, whole blood was collected from an adult Vietnamese cynomolgus macaque (male). In addition, for population analysis of *Mafa-B* alleles, peripheral blood samples were collected from 33 unrelated and healthy cynomolgus macaques of Vietnamese origin, which were housed in the South China Primate Research & Development Center (Guangdong, China), and peripheral blood mononuclear cells (PBMCs) were isolated.

### Pacbio HiFi library construction and sequencing

We extracted 30 μg of high-quality genomic DNA from white blood cells of the male cynomolgus macaque using blood and cell culture DNA kits (QIAGEN). Double-stranded DNA was fragmented, and the size distribution of the sheared DNA was characterized using the DNA 12,000 kit on the Agilent 2100 BioAnalyzer System. DNA fractions of approximately 15 kb were size selected for sequencing. PacBio-CCS sequencing libraries were prepared using the SMRTbell Template Prep Kit v.1.0 (Pacific. No. 100-259-100), according to the manufacturer’s protocol. Four SMRT flow cells were run on the PacBio Sequel II System with the Sequel Sequencing Kit 3.0 chemistry (Pacific Biosciences Ref. No.101-500-400 and 101-427-800) at BGI-Qingdao.

### Genome assembly

We used the Unanimity CCS software with the default parameter (–min-passes 3) to process the raw data into HiFi reads (https://github.com/pacificbiosciences/unanimity). Hifiasm (v0.12; -r1- × 0.9-y0.2) and Wtdbg2 (v2.3; -p23-E2S4-s0.05-L5000-X50; -j1500) software tools were used for de novo assembly of the generated HiFi reads [66, 67]. Hifiasm assembly was used to perform an all-versus-all pairwise alignment. Subsequent error correction was applied to remove most sequencing errors. Hifiasm tends to retain as much genome sequence information as possible, especially when dealing with complex regions; therefore, heterozygous variation information is retained [66]. Error-corrected reads were used to generate a draft genome. In addition, we assembled two independent haplotypes to phase complete MHC and KIR haplotypes by processing HiFi reads with hifiasm (v0.12; -r1- × 0.9-y0.2) in the same Vietnamese cynomolgus macaque [66]. The genome assembled by wtdbg2 was generated by directly assembling the raw data and then generating consensus reads through intermediate assembly outputs without eliminating sequencing errors [67]. After considering the results of the hifiasm and wtdbg2 assembly tools, the hifiasm-assembled genome was selected for subsequent genome annotation because the hifiasm assembly was more complete than the wtdbg2 assembly (3.65 Gb; Table 1) and more favorable for assembling complete *MHC* and *KIR* regions.

**Table 1:** Statistics of cynomolgus macaque genome assemblies

	Hifiasm	Wtdbg2
Total number (#)	2,468	1,397
Total length (bp)	3,650,970,991	2,729,663,640
Gap(bp)	0	0
Average length (bp)	1,479,324	1,953,947
N50 length (bp)	12,142,537	13,770,693
N90 length (bp)	1,808,563	2,371,347
Maximum length (bp)	56,411,848	50,183,256
Minimum length (bp)	11,420	1,385
GC content	41.16%	40.99%

Hifiasm (v0.12; -r1- × 0.9-y0.2) and Wtdbg2 (v2.3; -p23-E2S4-s0.05-L5000-X50;-j1500) software tools were used for de novo assembly with the generated HiFi reads

### Capture of MHC and KIR genomes

The contiguous *MHC* region was constructed in three steps. First, coding sequences (CDS) of the *MHC* genes (humans, rhesus and cynomolgus macaques) were downloaded from the Immuno Polymorphism Database (IPD)-NHMHC (version 3.4.0.0) and IPD-HLA (version 3.39) databases and aligned to the assembled Hifiasm genome using BLAST (v2.2.26) with an alignment length threshold of 500 bp. Second, we used BLAST (v2.2.26) to perform a collinear comparison of the human *MHC* sequences (chromosome 6:28,510,021–33,480,578) and the candidate *MHC* contigs obtained above. Third, the *MHC* CDS of cynomolgus monkeys were collinear compared with contig utg000348l (which represents the assembled MHC cluster) using BLAST (v2.2.26). The collinearity block length threshold was set to 200 bp. For *KIR* region analysis, CDS of *KIR* genes (humans, rhesus and cynomolgus macaques) from the IPD-NHKIR (version1.3.0.0) and IPD-KIR (version 2.10.0) databases were aligned to the assembled genome using BLAST (v2.2.26). The alignment length threshold was set at 100 bp.

### Gene annotations

Gene annotations included four aspects: repetitive sequence annotation, gene structure annotation, gene function annotation, and non-coding RNA (ncRNA) annotation (Additional file 1: Fig. S1). Two methods were used for repetitive sequence annotation: homology-based and de novo. We used RepeatMasker (v4.0.6) software (http://repeatmasker.org/) for homologous annotation based on Repbase (release 21.01) (http://www.girinst.org/repbase). Based on the sequence alignment of the genome itself, we used RepeatModeler (v2.0.1) [68], Piler (v1.0) [69] and RepeatScout (v1.0.6) [70] for gene annotations. Based on the characteristics of the repeat sequence, we used TRF (v4.07b) [71] and LTR-FINDER (v1.0.7) [72] for de novo annotation. Three sources of gene structure annotation were used: homolog annotation, de novo prediction, and transcript annotation. For homolog annotation, we selected protein sequences of six different species (*Homo sapiens*, *Macaca fascicularis*, *Monodelphis domestica*, *Mus musculus*, *Otolemur garnettii*, and *Pan troglodytes*) and compared them with the assembled genome using the software tool Genewise (v2.4.1) [73]. De novo prediction was performed using the software tools Augustus (v3.2.3) [74] and Genscan (v1.0) [75]. We then used a combination of the software tools Pasa (v.2.0.2) + Transdecoder (v.3.0.1) [76, 77] for transcript annotation. Finally, EVM (v1.1.1) [78] software was used to integrate the above-mentioned evidence sets and to filter out genes based on the following conditions: a gene has only one type of evidence supported by de novo prediction, the CDS length is short (< = 150 bp), and the overlap length ratio with TE is less than 0.2. The completeness of gene structure annotations was evaluated using BUSCO (v3.0.2), utilizing the Vertebrata odb9 set of 2,586 genes. Protein sequences obtained by gene structure annotation were compared with protein databases (SwissProt/TrEMBL (Release 2020_03, June 17, 2020) [79], KEGG (Release 94.2, June 1, 2020) [80], and InterPro (Release 80.0, June 18, 2020) [81] for functional annotation. For ncRNA annotations, we used tRNAscan-SE (v3.0) [82] software to search for tRNA sequences in the genome. Since rRNA is highly conserved, we used the rRNA sequences in the Rfam (v12.0) database as reference sequences to search for rRNA in the assembled genome by comparison with the RNAmmer (v1.2) tools [83]. In addition, the Rfam (v12.0) database and Infernal (v1.1) software [84] were used to predict the miRNA and snRNA sequences in the assembled genome.

We annotated the *MHC* genes using the previously annotated human *MHC* (NC_000006.12, BA000025.2), rhesus macaque *MHC* (NC_041757.1, AC148659-AC148717, AB128049.1), and cynomolgus macaque *MHC* (NC_022275.1) regions. Manual annotation was uniformly performed on the sequences. We used Blastn (v2.12.0, NCBI) to confirm the documented genes within the genomic sequences. Confirmed genes from this newly assembled genome were identical to previously assembled cynomolgus macaque cDNA sequences from the database or were orthologs to documented human or rhesus macaque genes. ncRNA and small nucleolar RNA (snoRNA) sequences were annotated based on human *MHC* sequences, whereas pseudogenes were defined as nonfunctional copies of reported genes with their coding regions disrupted by premature stopcodons and/or frameshift mutations. We annotated the *KIR* genes using exon sequences of rhesus and cynomolgus macaques from the IPD-NHKIR database (Release 1.3.0.0). The confirmed *Mafa-A/AG/B* and *Mafa-KIR* gene sequences were submitted to IPD to receive official designation [85, 86].

### RNA extraction, cDNA cloning, and sequencing

We used E.Z.N.A.™ Blood RNA Kits (OMEGA Bio-tek) to extract total RNA from PBMC samples of 33 unrelated and healthy cynomolgus macaques of Vietnamese origin. cDNA was synthesized using the PrimeScriptTM II 1st Strand cDNA Synthesis Kit (TaKaRa Bio, Kusatsu, Japan). For the specific amplification of ten *Mafa-B* loci, locus-specific primer sets were used to amplify exons 2 and 3. The forward primers were located in exons 1 or 2, and the reverse primers were located in exons 3, 4, or 5. The polymerase chain reaction (PCR) cycle conditions consisted of a denaturation process for 5 min at 95 °C, followed by 34 cycles at 95 °C for 30 s, 58 °C to 64 °C for 30 s, 72 °C for 25–50 s, and a final step at 72 °C for 10 min (Additional file 1: Table S1). PCR was performed in a 50 µL reaction mixture using Green *Taq* DNA mix (Vazyme). The PCR products were purified and ligated to the pMD19-T vector (TaKaRa). Ligations were transformed into Escherichia coli DH5α competent cells. Approximately 10–50 clones were selected for each *Mafa-B* amplicon from each animal and sequenced on an automatic DNA sequencer (ABI3730XL) by a service provider (Tsingke, Guangzhou, China). Nucleotide sequences of cDNAs were analyzed using SeqMan (DNASTAR, Madison, WI, USA) [87] and aligned using SnapGene 4.1.9(GLS Biotech, https://www.snapgene.com) and the Clustal W program (BioEdit) [88]. When a sequence was identical in at least three clones, it was considered an allele. These sequences were then submitted to GenBank for accession numbers.

## Results

### De novo assembly of the cynomolgus macaque genome

A total of 98.2 Gb of HiFi reads were obtained, with an average subread length of approximately 14 kb. Long-read assemblies (30 ×) of this cynomolgus macaque genome were generated using Hifiasm and Wtdb2 and yielded total sizes of 3.7 Gb and 2.7 Gb, respectively. The N50 sizes of contigs reached 12.1 Mb and 13.7 Mb, respectively; and the overall GC contents of the two assemblies were 41.16% and 40.99% (Table 1). Gene structures were annotated using EVM and predicted 31,606 genes, of which 81.41% were considered to be functional (Additional file 1: Tables S2, S3 and Fig. S2). BUSCO evaluation showed that 91.60% of the complete genes were fully annotated (Additional file 1: Table S4). In addition, ncRNA sequences were annotated in the cynomolgus macaque genome (Additional file 1: Table S5). This genome contained 49.23% repeat sequences that could be classified into different subtype elements, of which the majority represented long interspersed nuclear elements (LINEs) (Additional file 1: Fig. S3 and Tables S6, S7). In addition, two independent haplotypes of this cynomolgus macaque genome assembled with Hifiasm yielded total sizes of 3.1 Gb and 2.9 Gb, respectively, with N50 contigs of 16.9 Mb and 15.0 Mb (Additional file 1: Table S8).

### Physical mapping of the cynomolgus macaque *MHC* cluster

**Fig. 1:**
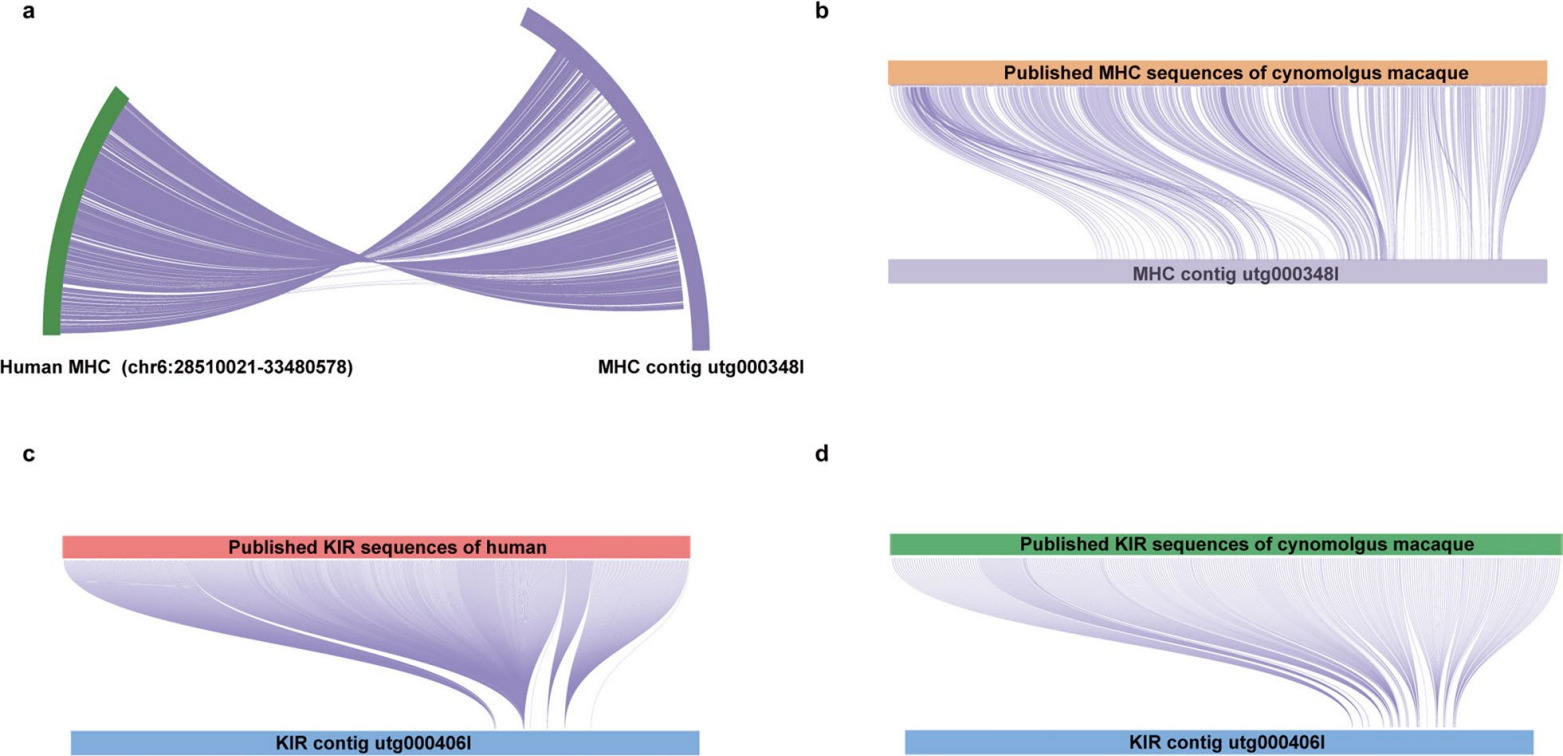
Collinearity between cynomolgus macaque contigs (utg000348l and utg000460l) and published *MHC* and *KIR* sequences, respectively. **a** BLAST (v2.2.26) was used to perform a collinear comparison of the human *MHC* sequences (chr6: 28,510,021–33,480,578) and the candidate MHC contig utg000348l. The left side of the figure represents the human *MHC* sequence (green), and the right side of the figure represents the sequence of the candidate MHC contig utg000348l (purple). **b** The *MHC* CDS sequences of cynomolgus macaques downloaded from the IPD were collinearly compared with the candidate MHC contig utg000348l using BLAST (v2.2.26). The top of the figure represents the *MHC* CDS sequences of cynomolgus macaques downloaded from the IPD (orange), and the bottom of the figure represents the sequences of the candidate MHC contig utg000348l (purple). **c** The *KIR* CDS sequences of human downloaded from the IPD were collinearly compared with the candidate KIR contig utg000460l using BLAST (v2.2.26). The top of the figure represents the *KIR* CDS sequences of human downloaded from the IPD (pink), and the bottom of the figure represents the sequences of the candidate KIR contig utg000460l (blue). **d** The *KIR* CDS sequences of cynomolgus macaques downloaded from the IPD were collinearly compared with the candidate KIR contig utg000460l using BLAST (v2.2.26). The top of the figure represents the *KIR* CDS sequences of cynomolgus macaques downloaded from the IPD (green), and the bottom of the figure represents the sequences of the candidate KIR contig utg000460l (blue)

Contig utg000348l (total length 8,094,345 bp) displayed collinearity with the humans (*MHC* region on chromosome 6) and cynomolgus macaque *MHC* sequences from the IPD database (Fig. 1a, b). It was defined as harboring the complete *MHC* region, including all class I and II genes (Fig. 1a, b).

**Fig. 2:**
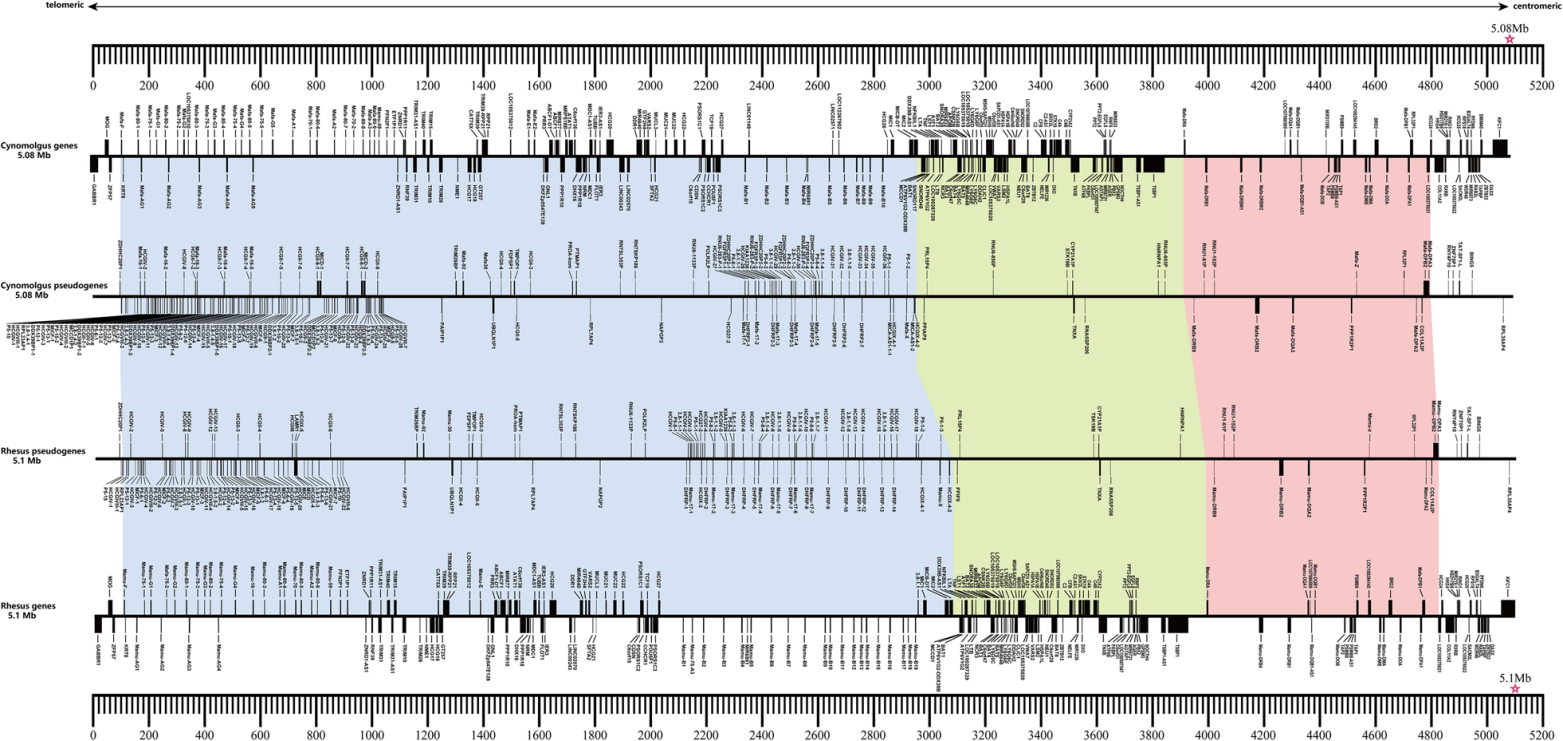
Linear representation of the cynomolgus and rhesus macaque *MHC*. The position and name of each gene in the MHC region are indicated above or below the horizontal line according to the convention, and in concordance with the forward or reverse orientation of a gene, respectively. The gene content of this cynomolgus macaque *MHC* in contig utg000348l was established by comparison with the rhesus macaque *MHC* (Daza-Vamenta et al., 2004; Jerzy K Kulski et al., 2004). For the cynomolgus and rhesus macaque MHC region, the positions of the pseudogenes are indicated on a separate horizontal line. Between both the cynomolgus and the rhesus macaque, the class I region ranges from *MHC-F* to *MIC2* (blue), the class III region ranges from *PPIAP9* to *RNU6-603P* (green), and the class II region ranges from *MHC-DRA* to *MHC-DPA3* (pink). There are some genes followed by a number (such as “−1”, “−2” and “−3”) to distinguish between different copies of the gene. The cynomolgus genes (top) and the rhesus genes (bottom) are scaled in kilobase pairs

This cynomolgus macaque *MHC* region in contig utg000348l spans 5.08 Mb, which is similar to the 5.1 Mb *MHC* region defined on the reference genome of rhesus macaques, considering the same start and end positions (Fig. 2) [11]. This contiguous region contained 453 genes that were annotated from *GABBR1*(located telomeric to the extended class I region) to *KIFC1* (located at the end of the extended class II region). Of these genes, 169 were predicted to be functional, 53 were classified as ncRNA, 5 genes were classified as snoRNAs, and the remaining 226 genes were classified as pseudogenes (Additional file 2: Table S9). Overall, a high level of conserved synteny was observed for cynomolgus and rhesus macaque *MHC* clusters with respect to functional genes as well as many pseudogenes. This assembled cynomolgus macaque *MHC* region shows an extension in size when compared to the *HLA* region of the human reference genome (hg38). This radical difference in size is the result of significant expansions within the macaque *MHC-A* and *-B* regions. The overall gene content in class II and III regions of this cynomolgus macaques overlaps with that of humans to a large extent. Five protein-coding genes (*HLA-C, BTNL2, HLA-DQA2, HLA-DQB2,* and *HLA-DRB5*) that were found in the *HLA* region had no orthologs in this cynomolgus macaque. Identical genes were defined in the *MHC* clusters of the two macaque species, except for the *SMIM40* gene, which was only identified in this cynomolgus macaque.

### Characteristics of the cynomolgus macaque *MHC* class I region

The composition and organization of the *MHC* region in contig utg000348l are considerably parallel between humans and macaques to a great extent. An exception was found for class I genes. In macaques, remodeling by 'birth and death' evolution resulted in an expansion of the number of class I genes, most likely in response to environmental pathogens. Even though a substantial fraction of *MHC* class I genes feature high conservation and homology, the number of genes with classical and non-classical characteristics within the *MHC* class I region differs extensively between humans and the two macaque species.

**Fig. 3:**
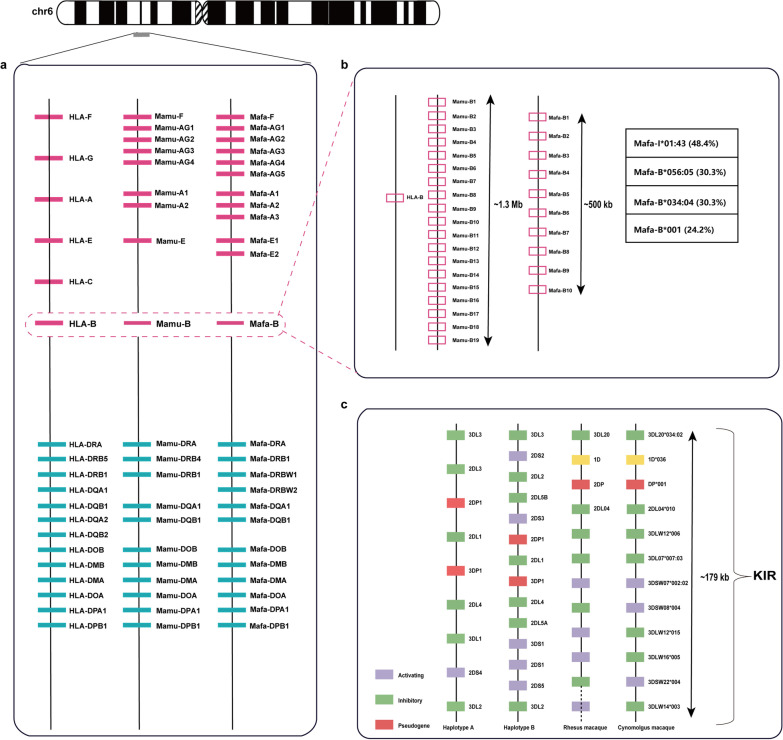
Comparative genomic map of the *MHC* and *KIR* region in human, rhesus and cynomolgus macaque. **a** Comparative genomic map of the protein-coding *MHC* genes in human (*HLA*), rhesus macaque (*Mamu*) and cynomolgus macaque (*Mafa*). Red and blue boxes indicate *MHC* class I and class II genes, respectively. Distances between genes are not scaled. **b** Comparative genomic map of the *HLA-B*, *Mamu-B* and *Mafa-B* regions (contig utg000348l). Distances between genes are not scaled. The table on the right shows the *Mafa-B* alleles with the highest frequency in our study (frequencies in between brackets). **c** Comparative genomic map of the *KIR* genes in human (haplotype A and B), rhesus and cynomolgus macaque (contig utg000460l). Distances between genes are not scaled. Purple represents activating *KIR* genes, green represents inhibitory *KIR* genes, pink represents pseudogenes, *and KIRID* genes are shown in yellow. Due to the high diversity of *KIR* genes in rhesus macaques, and the variable copy number of *KIR* genes per haplotype, the specific names of the *KIR* genes in rhesus macaques are not indicated in the figure

The *Mafa-A* region was subjected to duplication events, with three *Mafa-A* genes located in this assembled MHC region (Figs. 2, 3a). Five copies of *Mafa-AG* genes were identified as functional genes in this assembled MHC region (Figs. 2, 3a). Two copies of *Mafa-E* located close to each other were detected (Figs. 2, 3a). Six pseudogenes (*Mafa-59, Mafa-70, Mafa-92, Mafa-75, Mafa-80,* and *Mafa-30*) were identified as orthologs of human pseudogenes (Additional file 2: Table S9).

This assembled MHC region containing ten *Mafa-B* genes spans approximately 500 kb in contig utg000348l (Figs. 2, 3b). Of the ten *Mafa-B* genes, one has a stopcodon within exon 1 that presumably inactivates the gene. In comparison, 19 different *Mamu-B* genes were defined in the reference rhesus macaque MHC genome, 14 of which may encode proteins [11]. In addition, we detected a *Mafa-I* gene in this assembled MHC region (Fig. 2 and Additional file 2: Table S9). The *I* gene is the result of a duplication of the *B* gene and is also present in rhesus macaques [89].

In macaques, exon 1 of the *MHC-B* genes can contain one or two start codons. In this assembled MHC region, we observed that *Mafa-B1*, *Mafa-B6*, and *Mafa-B8* had two start codons, of which the first was out-of-frame and most likely the second was used as the start codon. Three other genes, *Mafa-B2*, *Mafa-B3*, and *Mafa-B5*, had two in-frame start codons, with the second ATG located at amino acid position 4 in the coding region (ATGCGGGTCATG). Only one start codon was identified in *Mafa-B4*, *Mafa-B7*, *Mafa-B9*, and *Mafa-B10*.

Overall, the majority of the genes present in this MHC region are conserved in humans, rhesus macaques, and cynomolgus macaques. Differential selective conditions, such as pathogen encounters, might have driven this extensive expansion observed in the macaque MHC class I region, which was not observed in humans.

### Transcription status of the ten identified *Mafa-B* genes in a panel of Vietnamese cynomolgus macaques

To elucidate which of the ten *B* genes identified on this Vietnamese cynomolgus *MHC* haplotype in contig utg000348l (Figs. 2, 3b) may encode a functional molecule and to identify high-frequency alleles, we performed *Mafa-B* locus-specific amplification in a cohort of 33 Vietnamese cynomolgus macaques (Additional file 2: Table S10). A total of 6,437 clones were sequenced, and 5,351 *Mafa-B* cDNA sequences were acquired. In the panel of 33 animals, we have identified 92 *Mafa-B* sequences (OK486180-OK486272). Most of these sequences contain only partial exons 2 and 3. Sixty-five of the sequences may represent novel alleles, and 27 of the sequences were identical to those reported previously in the IPD-MHC database [90]. Our results showed that the *B2, B3, B5,* and *B6* genes might be functional genes and are transcribed at abundant levels (Additional file 2: Table S11). Amplification with *B2* locus-specific primers resulted in 22 of the 33 cynomolgus macaques detecting a Mafa-I*01-like transcript. For *B3* locus-specific amplification, Mafa-B*056 was observed in 14 animals. At the *B5* locus, Mafa-B*034 was observed in 11 individuals, and our study showed a distribution frequency of 24.2% (8/33) for Mafa-B*001 at the *B6* locus. Furthermore, for the *B7* locus, Mafa-B*079 and Mafa-B*017 were detected in 6 and 2 animals, respectively. For *B1, B4,* and *B9* locus-specific amplification, we observed no transcripts (*B1* and *B4*) or alleles with low transcription levels (*B9*).

### Characteristics of the cynomolgus macaque *MHC* class II regions

The MHC class II regions (*MHC-DRA, -DQA1, -DQB1, ­DOB, -DMB, -DMA, -DOA, -DPA1* and *-DPB1*) are well conserved in humans and macaque species, except for the *MHC-DRB, MHC-DQA2*, and *MHC-DQB2* genes [91]. Five *DRB* genes were defined in this assembled MHC region of contig utg000348l, accompanied by one *DQA* gene and one *DQB* gene (Additional file 2: Table S9). Of these genes, *DRB2* and *DRB9* were pseudogenes. In rhesus macaques, the *MHC* class II region is similar to that in this cynomolgus macaques. In humans, the number of *DRB* genes present per haplotype ranged from one to four, and two *DQA* genes and two *DQB* genes could be identified [92]. The order of the genes within the human and macaque MHC class II regions was nearly identical.

### Genomic mapping of the cynomolgus macaque *KIR* gene region

Human and cynomolgus macaque *KIR* sequences from the IPD-KIR database displayed collinearity with contig utg000460l (total length: 1,114,979 bp) (Fig. 1c, d). This contig comprised the complete *KIR* haplotype, ranging from 765,301 to 944,995, thereby spanning a total length of 179,694 bp.

This assembled *KIR* region in contig utg000406l comprises 12 *KIR* genes, and the sequence similarity of the coding regions reached 98% to 100% compared to the cDNA references. This constructed *KIR* region was flanked by the *LILRA6* and *FCAR* genes, indicating that one complete *KIR* haplotype was assembled. All 12 *KIR* genes were organized in a head-to-tail arrangement and tightly clustered within 179 kb (Fig. 3c and Additional file 2: Table S12). The length of a single *KIR* gene varies from 8.5 to 15.1 kb. Three *KIR* sequences have been reported previously, whereas the remaining nine alleles were novel and received official designations. This assembled centromeric region comprises three genes, including the framework gene, *Mafa*-*KIR3DL20*, and a pseudogene, *Mafa-KIRDP*. The *Mafa-KIR1D* encodes receptors with a single extracellular domain and is thereby distinct from human lineage III *KIR* [45]. A *KIR2DL04* gene was identified in the telomeric region, which is conserved in humans and two macaque species [42]. In addition, eight lineage II *KIR* genes comprise approximately 115 kb of this assembled telomeric region, five of which are inhibitory genes, whereas three genes encode activating receptors.

### Characteristics of the cynomolgus macaque MHC class I region in two independent haplotypes

In haplotype 1, contig hltg000223l (total length 8,100,161 bp) displayed collinearity with *MHC* contig utg000348l (Additional file 1: Figs. S4, S5). In haplotype 2, three contigs showed collinearity with the *MHC* contig utg000348l (Additional file 1: Figs. S4, S5). They are as follows: h2tg000276l (total length 1,823,061 bp), h2tg000318l (total length 3,049,176 bp), and h2tg000147l (total length 6,336,003 bp). Of note, *MHC* class I genes were located on contigs h2tg000318l and h2tg000147l of haplotype 2 (Additional file 1: Fig. S6).

The assembled contig hltg000223l of haplotype 1 comprises five *Mafa-AG*, three *Mafa-A* and ten *Mafa-B* genes (Additional file 1: Fig. S7), ranging from 2,744,163 to 5,409,505 (Additional file 2: Table S13). The cDNA and genomic sequences of the *MHC* class I genes in contig hltg000223l were 100% identical to their counterparts on previously assembled MHC contig utg000348l, except for the *Mafa-A2* and *Mafa-B4* genes. The *Mafa-A2* gene in MHC contig utg000348l was named Mafa-A1*090:08:01:01 N and had an early stopcodon in exon 3. But the *Mafa-A2* gene in haplotype 1 has a deletion of two bases in exon 1, resulting in this *Mafa-A2* gene identical to allele Mafa-A1*090:04:02. Compared to the *Mafa-B4* gene (Mafa-B*109:30:01:01) in contig utg000348l, the *Mafa-B4* gene (Mafa-B*109:17:01:01) in haplotype 1 has one base deletion in exon 7.

Four *Mafa-AG*, two *Mafa-A* and sixteen *Mafa-B* genes were defined in haplotype 2 (Fig. S7), ranging from 892,631 to 3,028,576 in contig h2tg000318l and ranging from 40,057 to 659,990 in contig h2tg000147l (Additional file 2: Table S14). Of which, four *Mafa-AG*, two *Mafa-A* and seven *Mafa-B* genes were identical to the cDNA and genomic sequences of published *MHC* alleles. The remaining nine *Mafa-B* genes were novel and received official designations.

### Genomic mapping of the cynomolgus macaque* KIR* gene region in two independent haplotypes

In haplotype 1, contig hltg000304l (total length: 1,118,988 bp) displayed collinearity with *KIR* contig utg000406l (Additional file 1: Figs. S8 and S9). In haplotype 2, contigs h2tg000218l (total length 4,001,668 bp) and h2tg000293l (total length 633,137 bp) showed collinearity with the *KIR* contig utg000406l (Additional file 1: Figs. S8 and S9). However, all *KIR* genes are located on contig h2tg000293l of haplotype 2 (Additional file 1: Fig. S10).

Contig hltg000304l of haplotype 1 contains twelve *KIR* genes (Additional file 1: Fig. S11), ten of which are identical to the cDNA and genomic sequences of *KIR* genes on contig utg000406l. Two *KIR* genes (Mafa-KIR3DSW22*004:02 and Mafa-KIR3DLW14*003:02) have subtle base differences in the intron region with their counterparts located in contig utg000406l (Additional file 2: Table S15). All twelve *KIR* genes were clustered within 179 kb, ranging from 768,364 to 948,057 in contig hltg000304l of haplotype 1.

Eight *KIR* genes were defined in haplotype 2 (Additional file 1: Fig. S11), ranging from 339,299 to 464,543 in contig h2tg000293l (Additional file 2: Table S16). The sequence similarity of the coding region is 97% to 100% compared to the cDNA references. Two *KIR* genes have been reported previously, whereas the remaining six *KIR* alleles were novel.

Similar to the *MHC* class I region in macaques, the *KIR* gene region does not follow the standard organization. The gene content per haplotype displayed extensive diversity, as has been previously demonstrated for *MHC* and *KIR* haplotypes in cynomolgus and rhesus macaques [12–16, 41–48].

## Discussion

A contiguous and accurate cynomolgus macaque genome was de novo assembled using hifiasm and wtdbg2 with N50 contigs of 12.1 Mb and 13.7 Mb. Long-read sequencing was performed using the PacBio platform to characterize a complete reference cynomolgus macaque genome and reached a 30-fold coverage.

In the past decade, long-read sequencing techniques have developed rapidly, improving the continuity and quality of whole-genome assemblies. The assembled rheMacS increased the overall contiguity by 75-fold, closing 21,940 gaps of the previous assembly rheMac8 [62]. Compared with the rheMacS assembly, the cynomolgus macaque genome we assembled displayed less fragmentation (4741 vs. 2468/1397 contigs; 8.19 Mb vs. 12.14 Mb/13.77 Mb contig N50 length) [62]. The Mmul_10 genome assembly greatly improved the contiguity and completeness of the rhesus macaque reference genome with a contig N50 of 46 Mb [63]. Nonetheless, gaps still exist in the *Mamu-B* and *KIR* regions of the Mmul_10 assembly. Long-read data are particularly advantageous in resolving complex genomic regions, especially those with high repetitiveness and abundant GC content. The cynomolgus macaque genome assembled in this study contains two multi-gene families, *MHC* and *KIR* clusters, which are located on gap-free contigs. Currently, the representative genome of cynomolgus macaques is the chromosome-level assembly MFA1912RKSv2. The cynomolgus macaque genome we assembled with Hifiasm is 3.7 Gb, larger than the current cynomolgus monkey genome MFA1912RKSv2 (2.8 Gb) [64]. However, the contig N50 of the cynomolgus macaque genome assembled in this study was smaller than that of the MFA1912RKSv2 assembly. Despite this, the precise allelic-level annotation of the complex regions of *MHC* and *KIR* gene families with high content variability demonstrated that the genome assembly was accurate.

The *MHC* and *KIR* gene families experience various rounds of expansion and contraction facilitated by recombination events [13, 53]. In addition, both immune gene systems feature highly variable gene content and complicated sequence similarity, making the rapid genomic characterization of these multigenic families a challenge. Whereas short-read approaches hamper the complete and accurate characterization of these complex regions, SMRT sequencing platforms enable a comprehensive characterization of the *MHC* and *KIR* regions. Our cynomolgus macaque genome assembly was constructed from relatively long and high-accuracy contigs, allowing us to characterize complex regions that display extensive polymorphism and copy number variation. In addition, we phased complete MHC and KIR haplotypes in this cynomolgus macaque genome and comprehensively annotated genes located on the extended *MHC* class I and *KIR* region. The cDNA sequences of *MHC* class I and *KIR* genes on haplotype 1 are identical to their equivalents on contigs utg000348l and utg000406l, except for *Mafa-A2* and *Mafa-B4* genes. The two *Mafa-A2* genes have two bases difference in exon 1 and the two *Mafa-B4* genes have only one base difference in exon 7. Despite these slight base-level differences, but this demonstrates that the assembly strategy we have applied effectively phased diploid haplotypes without additional sequencing data. Together, four *Mafa-AG*, two *Mafa-A*, seven *Mafa-B* and two *KIR* genes in haplotype 2 were completely identical to previously described sequences of cynomolgus macaques. This further supports that our assemblies are precise at the allele level.

Different numbers of *MHC* class I genes were identified in this newly assembled cynomolgus macaque genome, it substantiates the diverse genetic content of this complex region. In rhesus macaques, one to six *Mamu-A* genes and up to nineteen *Mamu-B* genes can be present in a haplotype. A haplotype usually contains one or two major transcribed and up to five minor transcribed *Mamu-A* genes [13, 14]. For *Mamu-B* genes, one to six major transcripts and one to ten minor transcripts can be present per haplotype [13, 14]. This situation is similar in cynomolgus macaques. The number of *Mafa-A* and *Mafa-B* genes varies from one to six and one to seventeen per haplotype, respectively [93–96]. One to two major transcribed and up to five minor transcribed *Mafa-A* genes may be detected per haplotype [93–97]. A haplotype can comprise one to seven major transcribed and up to fifteen minor transcribed *Mafa-B* genes [93–97]. Our data illustrated that the number of *Mafa-A* and *Mafa-B* genes varies among haplotypes in this assembled cynomolgus macaque genome. The *Mafa-B* regions contain ten and sixteen *Mafa-B* genes in haplotype 1 and haplotype 2, respectively. Despite this, it represents only two haplotypes in cynomolgus macaque. One study showed that one detected *Mamu-B* haplotype matched the *Mamu-B* region in rhesus *MHC* published in 2004, with only eight *Mamu-B* transcripts observed, whereas no transcripts were detected for the other *Mamu-B* loci [11, 98]. This indicates that most *Mamu-B* genes are not transcribed. The high variability in gene copy number combined with the differential transcription levels in *MHC* class I genes highlight a different selective sweep occurring in their *MHC* class I repertoire in macaques. Although there is an evident discrepancy in the variation of *MHC* class I genes between the two widely used non-human primate animal models, many studies have demonstrated that a few conserved *MHC* class I genes in both macaque species may have evolved to fulfill important immune functions. These conserved genes may have fine-tuned their sequences in response to environmental pathogens [14, 93, 99]. Our previous studies have confirmed that ancient introgression occurs at the junction of the two species, as extremely high nucleotide sequence similarity was observed between Chinese rhesus macaques and cynomolgus macaques [59]. A comparison of rhesus and cynomolgus macaques as models of COVID-19 infection showed that both species responded similarly to severe acute respiratory syndrome coronavirus 2 (SARS-CoV-2) infection when challenged with SARS-CoV-2 [100]. The high rate of gene overlap and similar immune responses to infection will advance the widely use of cynomolgus macaques as preclinical animal models in biomedical research to study human diseases.

*KIR* genes are characterized by homology, duplication, and structural diversity in macaques, making this region increasingly complex. Studies of the *KIR* region in rhesus and cynomolgus macaques showed a differential number of *KIR* genes among different haplotypes and populations [48–52]. Characterization of the *KIR* transcriptome of 298 Indian rhesus macaques using SMRT sequencing yielded 112 unique *KIR* haplotype configurations, and each haplotype contains 4 to 17 different *KIR* genes [42, 48]. Based on published transcriptome data, a cynomolgus macaque haplotype contains 3 to 13 different *KIR* genes [48]. Our currently assembled cynomolgus macaque *KIR* haplotypes represent only two combinations of *KIR* genes and differ in gene content. Many *MHC* class I and class II genes have been shared in rhesus and cynomolgus macaques [14, 95, 101]. However, only a few *KIR* alleles were shared between the two macaque species. The occurrence of chromosomal recombination, point mutations, alternative splicing, and stochastic expression results in a large number of orthologous and species-specific *KIR* genes [53]. This indicates that different selective forces drive the evolution of the *KIR* system, as evidenced by differences in lineage expansion and haplotype configurations between rhesus and cynomolgus macaques. Our extended knowledge of the relatively high levels of species-specific *KIR* genes indicates considerable diversity and complexity, whereas the homologous genes shared between the two highly related macaque species reflect a common ancestry. A comprehensive overview of *KIR* genes is fundamental for the study of *KIRs*, which advances the study of using macaques as a model and facilitates future studies on the role of *KIRs* in immunogenetics. The extensive diversity of MHC class I molecules may have prompted the rapid selection of KIR molecules. This can be readily understood in the context of our understanding of the highly polymorphic MHC class I molecules in macaques, as MHC class I molecules are specific ligands for KIR molecules. Thus, the extreme complexity of macaque KIR molecules illustrates their coevolution with MHC ligands. Different sets of *KIR* and *MHC* genes have been associated with the progression of AIDS [102], hepatitis C [103], reproduction [104, 105] and hematopoietic stem cell transplantation [106]. A holistic analysis of *MHC* and *KIR* genes may contribute to a better understanding of immunogenetics by studying the complex functions of *MHC/KIR* pairs.

It was found that *Mafa-B* genes have the highest degree of duplication among the class I genes. We performed locus-specific amplification of ten *Mafa-B* loci in 33 cynomolgus macaques of Vietnamese origin. We identified four functional loci, *B2, B3, B5,* and *B6* at the transcriptome level, with Mafa-I*01, Mafa-B*056, Mafa-B*034, and Mafa-B*001 lineages displaying the highest frequencies. For most macaque *B* haplotypes, two or three genes are transcribed at substantial levels, which are thought to fulfill the classical *MHC* antigen-presenting function [93–97]. The highly frequent *I* gene has the characteristics of classical and non-classical genes and most likely executes a more specific function [107]. A previous study found that the peptide Gag QI9 was presented by Mamu-A1*001:01 and also presented by Mamu-B*056:01 [108]. In a rhesus macaque SIV infection model, Mamu-A1*001:01 was identified as a protective allele [109]. It would be interesting to test whether the highly frequent Mafa-B*56 lineage alleles that we identified, which are orthologs of the Mamu-B*56 lineage, can also present Gag QI9. For the *B11L* gene (at the *B6* gene), we identified several transcripts orthologous to *B11L* with a frequency distribution of 21.2% (7/33). The Mamu-B*001 allele confers resistance to CIA [29]. In our study, we found that the distribution frequency of Mafa-B*001 was 24.2% (8/33) at the *B6* locus. However, whether Mafa-B*001 is resistant to the CIA is currently unknown. Of the ten *B* loci, *B7, B8,* and *B10* genes have been reported to be pseudogenes [10]. However, for the *B7* locus, we detected alleles of the Mafa-B*079 and Mafa-B*017 lineages in 6 and 2 animals, respectively. Of these, Mafa-B*017:02 is homologous to Mamu-B*017:01. Mamu-B*017:01 controls SIV replication and disease progression [26]. The *B1, B4,* and *B9* genes were reported to have low transcription levels [93–97], and only three alleles (Mafa-B*180:02:01:02nov, Mafa-B*124:03:01:01nov, Mafa-B*021:07nov) were detected in the *B9* locus. The combinations I*01-B*056:05-B*034:04 (animal 1, 5, 6, 18, 21) and I*01-B*034:04-B11L*01 (animal 16) matched the *Mafa-B* region in contig utg000348l. In addition, the combinations B*105:01-B*156:01 (animal 10) and B*105:01-B*001:01 (animal 33) matched the *Mafa-B* region in haplotype 2.

However, our sample size was too small to include all *Mafa-B* alleles. Clone-based strategies are expensive and time-consuming, and amplification may not recover clones sufficiently, resulting in transcripts at low transcription levels that may not be identified. In addition, the sequence similarity between *MHC* genes makes the design of specific primers difficult, and a large number of primer pairs must be designed to enable comprehensive genotyping. Nevertheless, it may be a relatively effective PCR genotyping method to design primers to amplify amplicons spanning the highly conserved peptide-binding domain of *MHC* class I molecules [12]. A strategy was developed for *KIR* genotyping by designing primers based on highly conserved sequences in the D1 domain (most of the region), the D2 domain and the stem region (part of the region) [52]. Comprehensive *MHC* and *KIR* genotyping is important for better understanding the role of these polymorphisms in human disease models.

This study has limitations, as the *MHC* and *KIR* genomic regions analyzed here represent only one example in cynomolgus macaques. Therefore, additional haplotypes at the genomic level are required to obtain a comprehensive overview of the complexity of these immune regions in this species, as well as supplementary studies to generate the complete *MHC and KIR* genotypes and compare the detected genes to this currently assembled genome.

## Conclusions

We constructed full-length MHC and KIR regions in a Vietnamese cynomolgus macaque. There were 453 loci in the extended MHC region and 12 loci in the KIR cluster in this new reference cynomolgus macaque genome. The *Mafa-B* genes displayed the highest degree of duplication among *MHC* class I genes. We identified four functional *Mafa-B* loci in this cynomolgus macaque *MHC* region. The gene content of the MHC class I region and KIR region is highly variable between the two independent haplotypes in this assembly. Knowledge gained on the genetic organization of *MHC* class I and *KIR* genes in macaques contributes to the understanding of how the immune system evolved and lays the foundation for investigating NK cell responses in non-human primate models.

## Supplementary Information

**Additional file 1**: **Fig S1** Flow chart of gene annotations. **Fig S2** Statistics of functional annotations in the assembled cynomolgus macaque genome. A Venn diagram shows the overlap between the different programs used to calculate the functional annotations. Functional annotations were performed using Swissprot, KEGG, TrEMBL, and Interpro. **Fig S3** Distribution of the divergence rate of each type of the assembled cynomolgus macaque’s transposable elements (TEs). The divergence rate was calculated between the identified TEs in the genome by homology-based method and the consensus sequence in the Repbase. Different TEs are marked with different colors. **Fig S4** Collinearity analysis of MHC contig utg000348l and candidate MHC contigs in haplotype 1 and 2. We assembled two independent haplotypes by processing HiFi reads using hifiasm. The MHC contig utg000348l was aligned to assembled haplotype 1 and 2 using BLAST (v2.2.26). We obtained one candidate MHC contig and three candidate MHC contigs in haplotype 1 and 2, respectively. In haplotype 1, one contig, hltg000223l (purple), displayed collinearity with MHC contig utg000348l (gray). Some high-repetition areas have multiple comparisons, which are shown in the areas with dense lines plot. In haplotype 2, three contigs showed collinearity with the MHC contig utg000348l. They are as follows: h2tg000276l (pink), h2tg000318l (green), and h2tg000147l (only 3.05 Mb displayed here; blue). This figure shows only identity>0.95 and block>2000 bp. **Fig S5** Sequence alignment of MHC contig utg000348l with candidate MHC contigs in haplotype 1 and 2. The MHC contig utg000348l was aligned with four candidate MHC contigs (h1tg000223l, h2tg000276l, h2tg000318l, and h2tg000147l) in haplotype 1 and 2 using Minimap2 (v2.24; r1122). Contig hltg000223l (gray) in haplotype 1 is nearly identical to MHC contig utg000348l. In haplotype 2, h2tg000276l (red) and h2tg000318l (blue) have no significant overlap; h2tg000318l (blue) and h2tg000147l (pink) have significant overlap with over 200 kb, but there are significant sequence differences in the overlap region. There are clear insertions and deletions in all three contigs (h2tg000276l, h2tg000318l, and h2tg000147l). Purple arrows and purple lines on each contig indicate insertions (only insertions >500 bp are shown); black bold lines indicate deletions (deletions >500 bp are shown). Lines in red, green, and blue on contig utg000348l indicate base mutations. **Fig S6** Collinearity between cynomolgus macaque candidate MHC contigs and published sequences of MHC class I genes. The CDS sequences of MHC class I genes in cynomolgus and rhesus macaques downloaded from the IPD (purple) were collinearly compared with the four candidate MHC contigs hltg000223l, h2tg000276l, h2tg000318l and h2tg000147l using BLAST (v2.2.26). Contigs hltg000223l (green), h2tg000318l (pink) and h2tg000147l (blue) displayed collinearity with the CDS sequences of MHC class I genes in cynomolgus and rhesus macaques downloaded from the IPD (purple). The comparison results showed no MHC class I gene on contig h2tg000276l. **Fig S7** Linear representation of the cynomolgus macaque MHC genes in contig utg000348l and the two phased haplotypes. These MHC sequences in haplotype 1 and 2 were compared with the alleles in Immuno Polymorphism Database (IPD) to find the exons and introns of each allele. The novel sequences were received official designations. Distances between genes are not scaled. **Fig S8** Collinearity analysis of KIR contig utg000406l and candidate KIR contigs in haplotype 1 and 2. The KIR contig utg000406l was aligned to the assembled haplotype 1 and 2 using BLAST (v2.2.26). As a result, we obtained one candidate KIR contig and two candidate KIR contigs in haplotype 1 and 2, respectively. In haplotype 1, one contig, hltg000304l (blue), displayed collinearity with KIR contig utg000406l (gray). Some high-repetition areas have multiple comparisons, which are shown in the areas with dense lines plot. In haplotype 2, two contigs showed collinearity with the KIR contig utg000406l. They are as follows: h2tg000218l (only 330 kb displayed here; pink) and h2tg000293l (green). This figure shows only identity>0.95 and block>500 bp. **Fig S9** Sequence alignment of KIR contig utg000406l with candidate KIR contigs in haplotype 1 and 2. The KIR contig utg000406l was aligned with three candidate KIR contigs (hltg000304l, h2tg000218l, and h2tg000293l) in haplotype 1 and 2 using Minimap2 (v2.24; r1122). Contig hltg000304l (red) in haplotype 1 is nearly identical to KIR contig utg000406l. In haplotype 2, h2tg000218l (blue) and h2tg000239l (green) have no significant overlap. There are clear insertions and deletions in the two contigs (h2tg000218l and h2tg000293l). Black bold lines indicate deletions (deletions>500 bp are shown). Lines in red, green, and blue on contig utg000406l indicate base mutations. **Fig S10** Collinearity between cynomolgus macaque candidate KIR contigs and published KIR sequences. The KIR CDS sequences of cynomolgus and rhesus macaques downloaded from the IPD (purple) were collinearly compared with the three candidate KIR contigs hltg000304l, h2tg000293l and h2tg000218l using BLAST (v2.2.26). Contigs hltg000304l (pink) and h2tg000293l (blue) displayed collinearity with the KIR CDS sequences of cynomolgus and rhesus macaques downloaded from the IPD (purple). The comparison results showed that there was no KIR gene on h2tg000218l. **Fig S11** Linear representation of the cynomolgus macaque KIR genes in contig utg000406l and the two phased haplotypes. These KIR sequences in haplotype 1 and 2 were compared with the alleles in Immuno Polymorphism Database (IPD) to find the exons and introns of each allele. The novel sequences were received official designations. Distances between genes are not scaled. **Table S1** Locus-specific primers for the ten different Mafa-B loci. **Table S2** Statistics of gene structure annotations in the assembled cynomolgus macaque genome. **Table S3** Statistics of functional genes in the assembled cynomolgus macaque genome. **Table S4** BUSCO evaluation of the assembled cynomolgus macaque genome. **Table S5** Statistics of non-coding RNA genes in the assembled cynomolgus macaque genome.**Table S6** Statistics of repeats in the assembled cynomolgus macaque genome. **Table S7** Transposable elements (TEs) content in the assembled cynomolgus macaque genome. **Table S8** Statistics of phased haplotypes of cynomolgus macaque genome.

**Additional file 2**: **Table S9** Cynomolgus macaque MHC gene annotations in contig utg000348l. **Table S10** Description of the 33 Vietnamese cynomolgus macaques. **Table S11** Distribution of Mafa-B alleles at 10 Mafa-B locus. **Table S12** Cynomolgus macaque KIR gene annotations in contig utg000406l. **Table S13** Cynomolgus macaque MHC gene annotations in contig hltg000223l (haplotype 1). **Table S14** Cynomolgus macaque MHC gene annotations in contigs h2tg000318l and h2tg000147l (haplotype 2). **Table S15** Cynomolgus macaque KIR gene annotations in contig hltg000304l (haplotype 1). **Table S16** Cynomolgus macaque KIR gene annotations in contig h2tg000293l (haplotype 2).

## Data Availability

The third-generation long-read sequencing data, sequence of MHC contigs and KIR contigs have been deposited in NCBI under the project accession number PRJNA819149 and PRJNA847748. The sequences of *Mafa-A/Mafa-AG/Mafa-B/Mafa-KIR* obtained from this study have been submitted to GenBank under accession number MW809291-MW809293、MW809286-MW809290、MZ254652-MZ254661、MZ436149-MZ436163 respectively. The sequences of 92 *Mafa-B* alleles obtained from this study have been submitted to GenBank under accession number OK486180-OK486272. The rhesus macaque reference *MHC* sequence data was under accession number AB128049 (https://www.ncbi.nlm.nih.gov/nuccore/AB128049.1/), NC_041757.1(https://www.ncbi.nlm.nih.gov/nuccore/NC_041757.1/) and AC148659-AC148717. The sequence data of human reference *MHC* was under accession number NC_000006.12 (https://www.ncbi.nlm.nih.gov/search/all/?term=NC_000006.12) and BA000025.2(https://www.ncbi.nlm.nih.gov/nuccore/BA000025.2/). The sequence data of cynomolgus macaque reference *MHC* was under accession number NC_022275.1 (https://www.ncbi.nlm.nih.gov/nuccore/NC_022275.1?report=genbank).

## Abbreviations

MHC: Major histocompatibility complex
KIR: Killer cell immunoglobulin-like receptor
COVID-19: Coronavirus disease 2019
*Mafa*: *Macaca fascicularis*
*Mamu*: *Macaca mulatta*
AIDS: Acquired immunodeficiency syndrome
HLA: Human leukocyte antigen
ONT: Oxford Nanopore Technologies
SMRT: Single-molecule real-time sequencing
CCS: Circular consensus sequencing
CDS: Coding sequences
IPD: Immuno Polymorphism Database
ncRNA: Non-coding RNA
EVM: EVidenceModeler
LINEs: Long interspersed nuclear elements
