# Supplementary material for: Long-read assembly of major histocompatibility complex and killer cell immunoglobulin-like receptor genome regions in cynomolgus macaque
Source: Biol Direct. 2022 Nov 29;17:36. doi: 10.1186/s13062-022-00350-w (PMC9707422; doi:10.1186/s13062-022-00350-w)
Supplement: Supplementary file 1 — Additional file 1: Fig S1 Flow chart of gene annotations. Fig S2 Statistics of functional annotations in the assembled cynomolgus macaque genome. A Venn diagram shows the overlap between the different programs used to calculate the functional annotations. Functional annotations were performed using Swissprot, KEGG, TrEMBL, and Interpro. Fig S3 Distribution of the divergence rate of each type of the assembled cynomolgus macaque’s transposable elements (TEs). The divergence rate was calculated between the identified TEs in the genome by homology-based method and the consensus sequence in the Repbase. Different TEs are marked with different colors. Fig S4 Collinearity analysis of MHC contig utg000348l and candidate MHC contigs in haplotype 1 and 2. We assembled two independent haplotypes by processing HiFi reads using hifiasm. The MHC contig utg000348l was aligned to assembled haplotype 1 and 2 using BLAST (v2.2.26). We obtained one candidate MHC contig and three candidate MHC contigs in haplotype 1 and 2, respectively. In haplotype 1, one contig, hltg000223l (purple), displayed collinearity with MHC contig utg000348l (gray). Some high-repetition areas have multiple comparisons, which are shown in the areas with dense lines plot. In haplotype 2, three contigs showed collinearity with the MHC contig utg000348l. They are as follows: h2tg000276l (pink), h2tg000318l (green), and h2tg000147l (only 3.05 Mb displayed here; blue). This figure shows only identity>0.95 and block>2000 bp. Fig S5 Sequence alignment of MHC contig utg000348l with candidate MHC contigs in haplotype 1 and 2. The MHC contig utg000348l was aligned with four candidate MHC contigs (h1tg000223l, h2tg000276l, h2tg000318l, and h2tg000147l) in haplotype 1 and 2 using Minimap2 (v2.24; r1122). Contig hltg000223l (gray) in haplotype 1 is nearly identical to MHC contig utg000348l. In haplotype 2, h2tg000276l (red) and h2tg000318l (blue) have no significant overlap; h2tg000318l (blue) and h2tg000147l (pink) have s [file 13062_2022_350_MOESM1_ESM.docx]

Additional file 1:


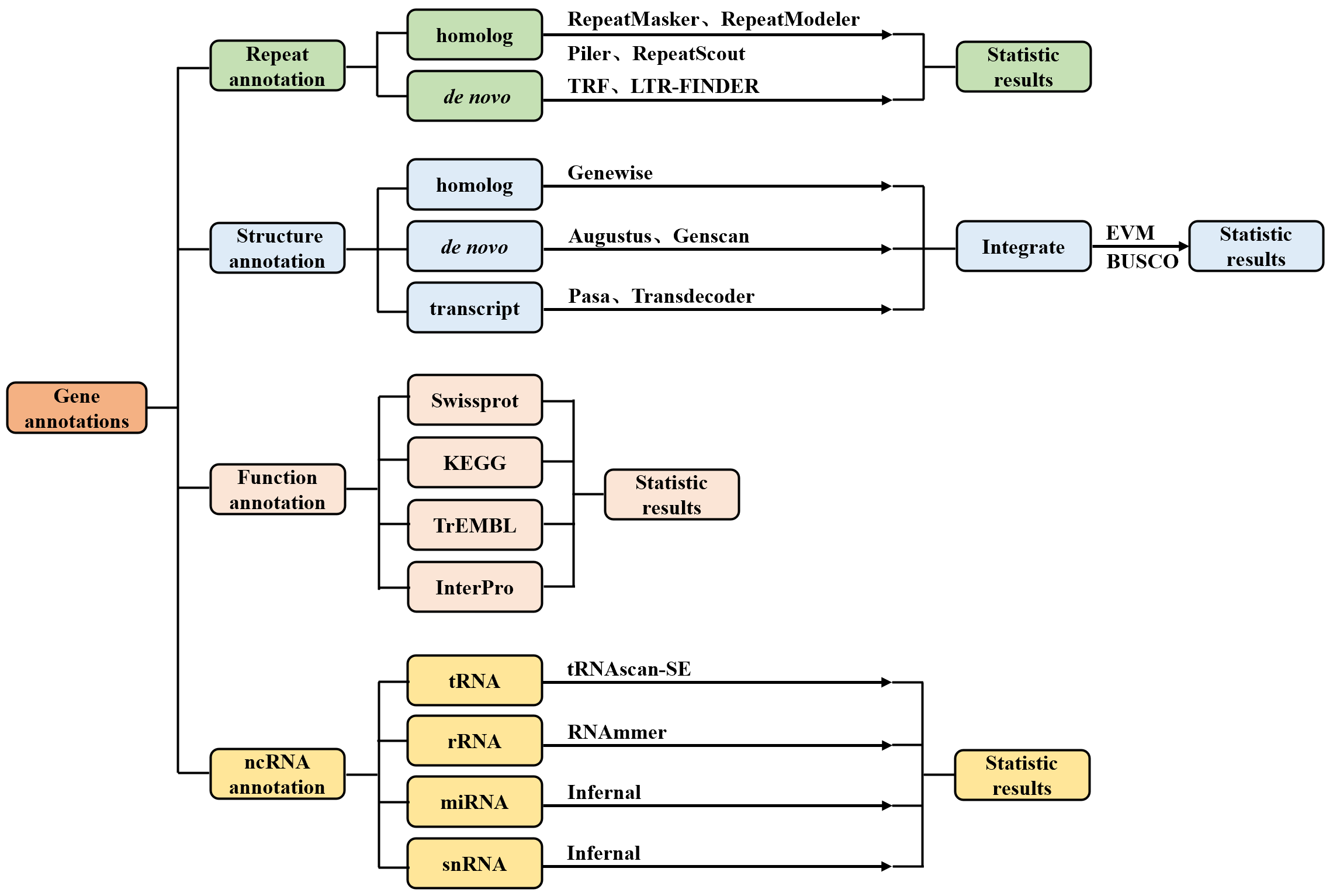


**Fig. S1 Flow chart of gene annotations.**


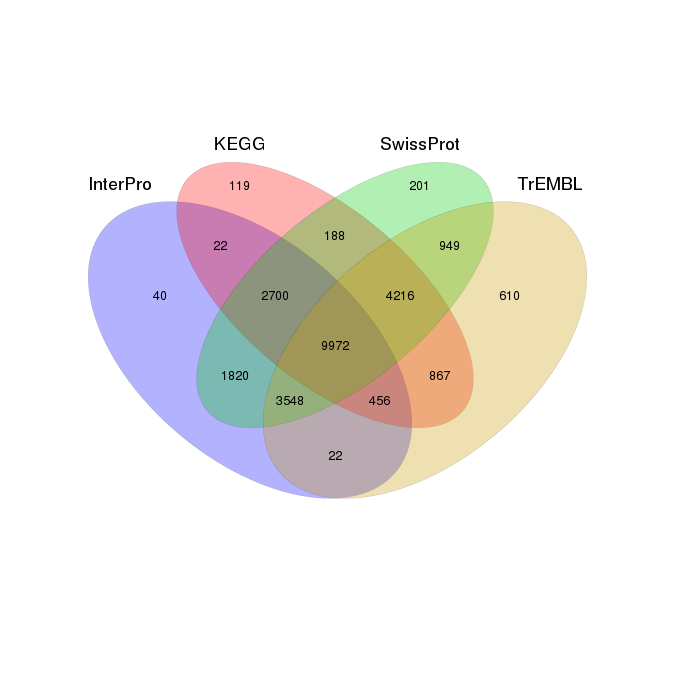


**Fig. S****2 Statistics of functional annotations in the assembled cynomolgus macaque genome.** A Venn diagram shows the overlap between the different programs used to calculate the functional annotations. Functional annotations were performed using Swissprot, KEGG, TrEMBL, and Interpro.


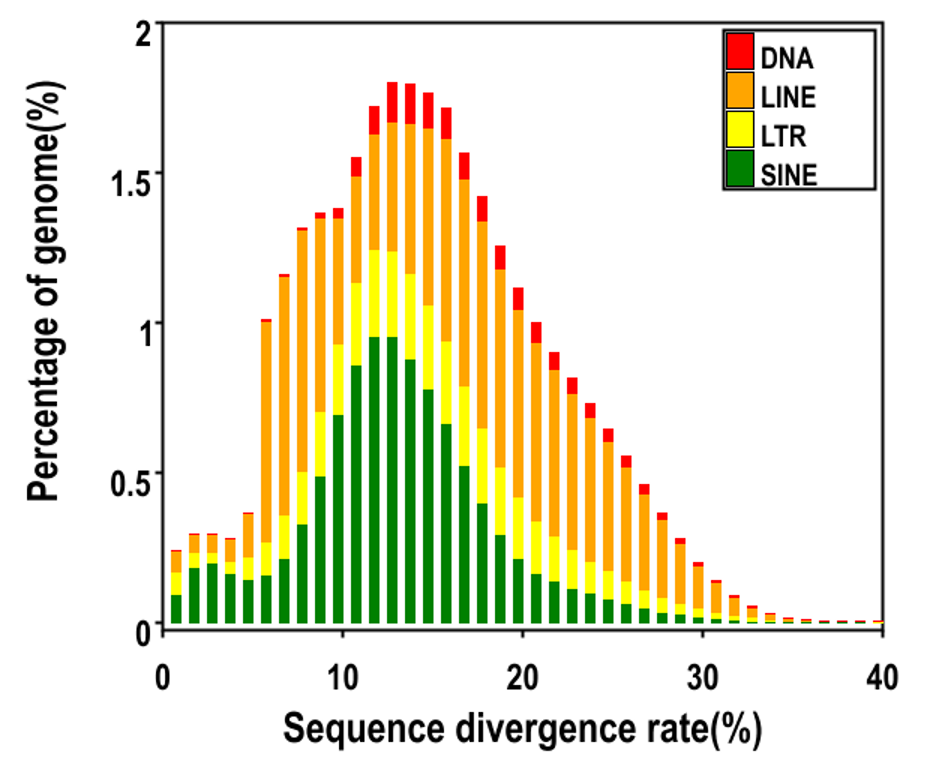


**Fig****. S3 Distribution of the divergence rate of each type of the assembled cynomolgus macaque’s transposable elements (TEs).** The divergence rate was calculated between the identified TEs in the genome by homology-based method and the consensus sequence in the Repbase. Different TEs are marked with different colors.


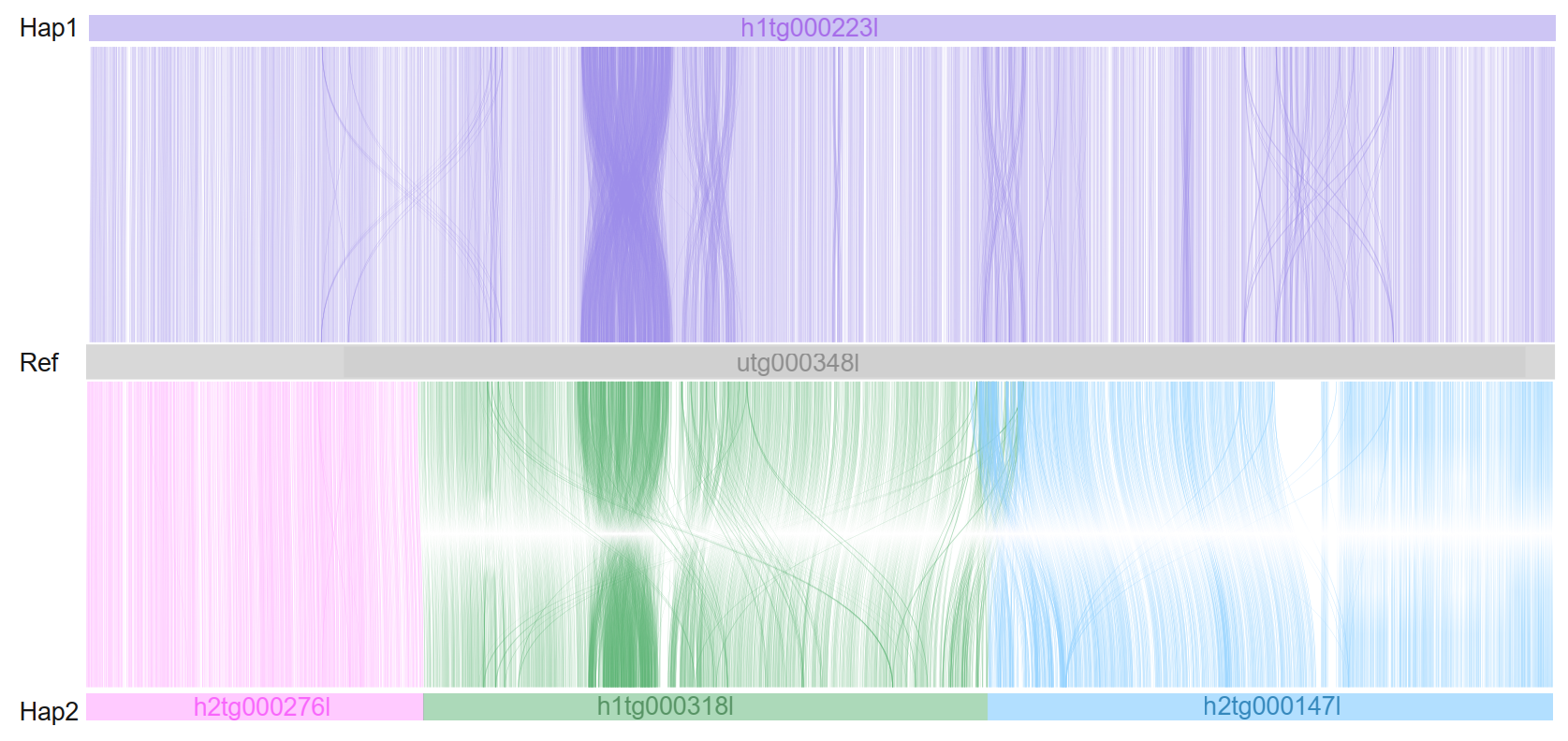


**Fig. S4 Collinearity analysis of MHC contig utg000348l and candidate MHC contigs in haplotype 1 and 2.** We assembled two independent haplotypes by processing HiFi reads using hifiasm. The MHC contig utg000348l was aligned to assembled haplotype 1 and 2 using BLAST (v2.2.26). We obtained one candidate MHC contig and three candidate MHC contigs in haplotype 1 and 2, respectively. In haplotype 1, one contig, hltg000223l (purple), displayed collinearity with MHC contig utg000348l (gray). Some high-repetition areas have multiple comparisons, which are shown in the areas with dense lines plot. In haplotype 2, three contigs showed collinearity with the MHC contig utg000348l. They are as follows: h2tg000276l (pink), h2tg000318l (green), and h2tg000147l (only 3.05 Mb displayed here; blue). This figure shows only identity>0.95 and block>2000 bp.


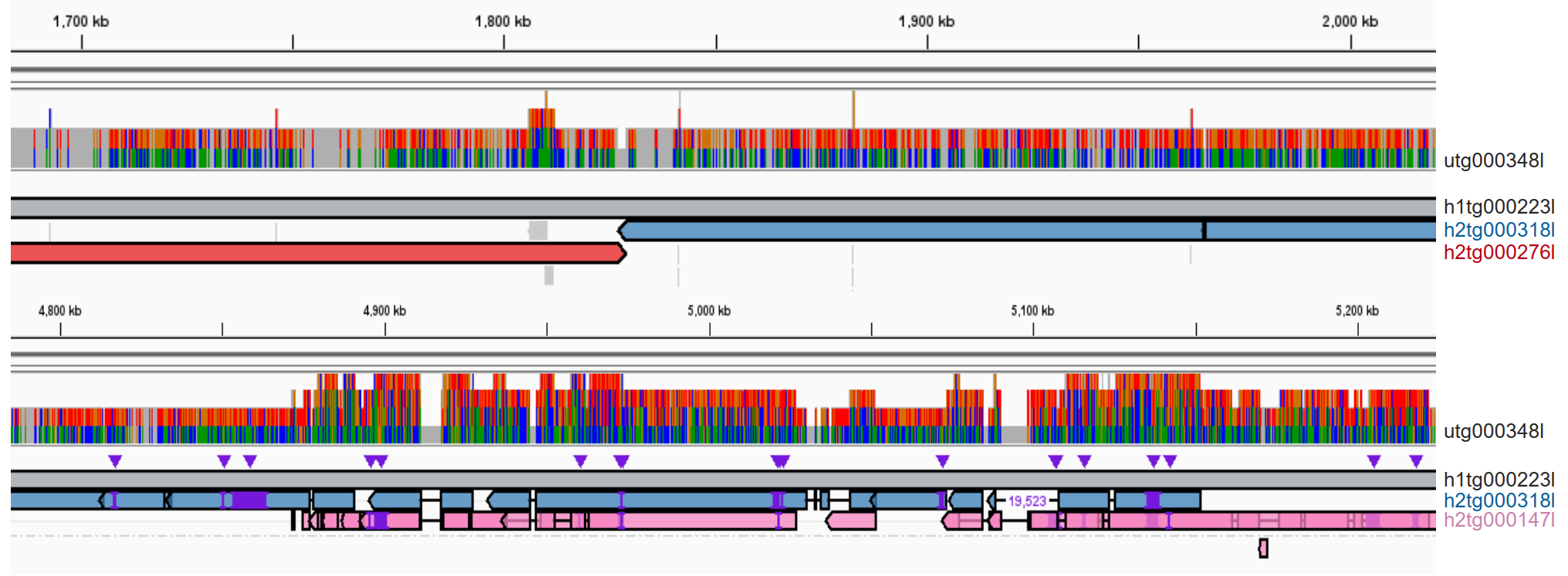


**Fig. S5** **Sequence alignment of MHC contig utg000348l with candidate MHC contigs in haplotype 1 and 2**. The MHC contig utg000348l was aligned with four candidate MHC contigs (h1tg000223l, h2tg000276l, h2tg000318l, and h2tg000147l) in haplotype 1 and 2 using Minimap2 (v2.24; r1122). Contig hltg000223l (gray) in haplotype 1 is nearly identical to MHC contig utg000348l. In haplotype 2, h2tg000276l (red) and h2tg000318l (blue) have no significant overlap; h2tg000318l (blue) and h2tg000147l (pink) have significant overlap with over 200 kb, but there are significant sequence differences in the overlap region. There are clear insertions and deletions in all three contigs (h2tg000276l, h2tg000318l, and h2tg000147l). Purple arrows and purple lines on each contig indicate insertions (only insertions >500 bp are shown); black bold lines indicate deletions (deletions >500 bp are shown). Lines in red, green, and blue on contig utg000348l indicate base mutations.


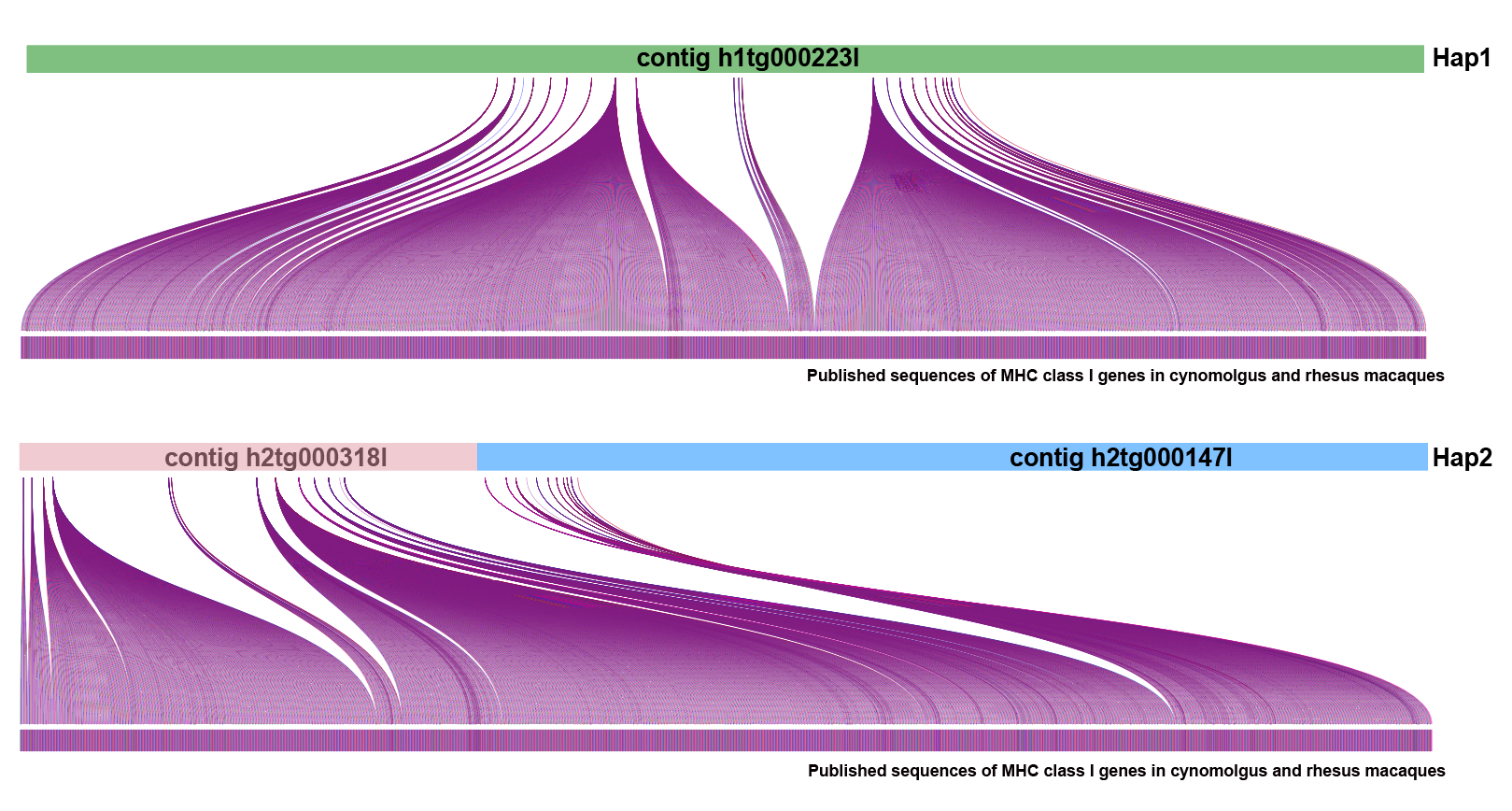


**Fig. S6 Collinearity between cynomolgus macaque candidate MHC contigs and published sequences of *MHC* class I genes.** The CDS sequences of *MHC* class I genes in cynomolgus and rhesus macaques downloaded from the IPD (purple) were collinearly compared with the four candidate MHC contigs hltg000223l, h2tg000276l, h2tg000318l and h2tg000147l using BLAST (v2.2.26). Contigs hltg000223l (green), h2tg000318l (pink) and h2tg000147l (blue) displayed collinearity with the CDS sequences of *MHC* class I genes in cynomolgus and rhesus macaques downloaded from the IPD (purple). The comparison results showed no *MHC* class I gene on contig h2tg000276l.

**
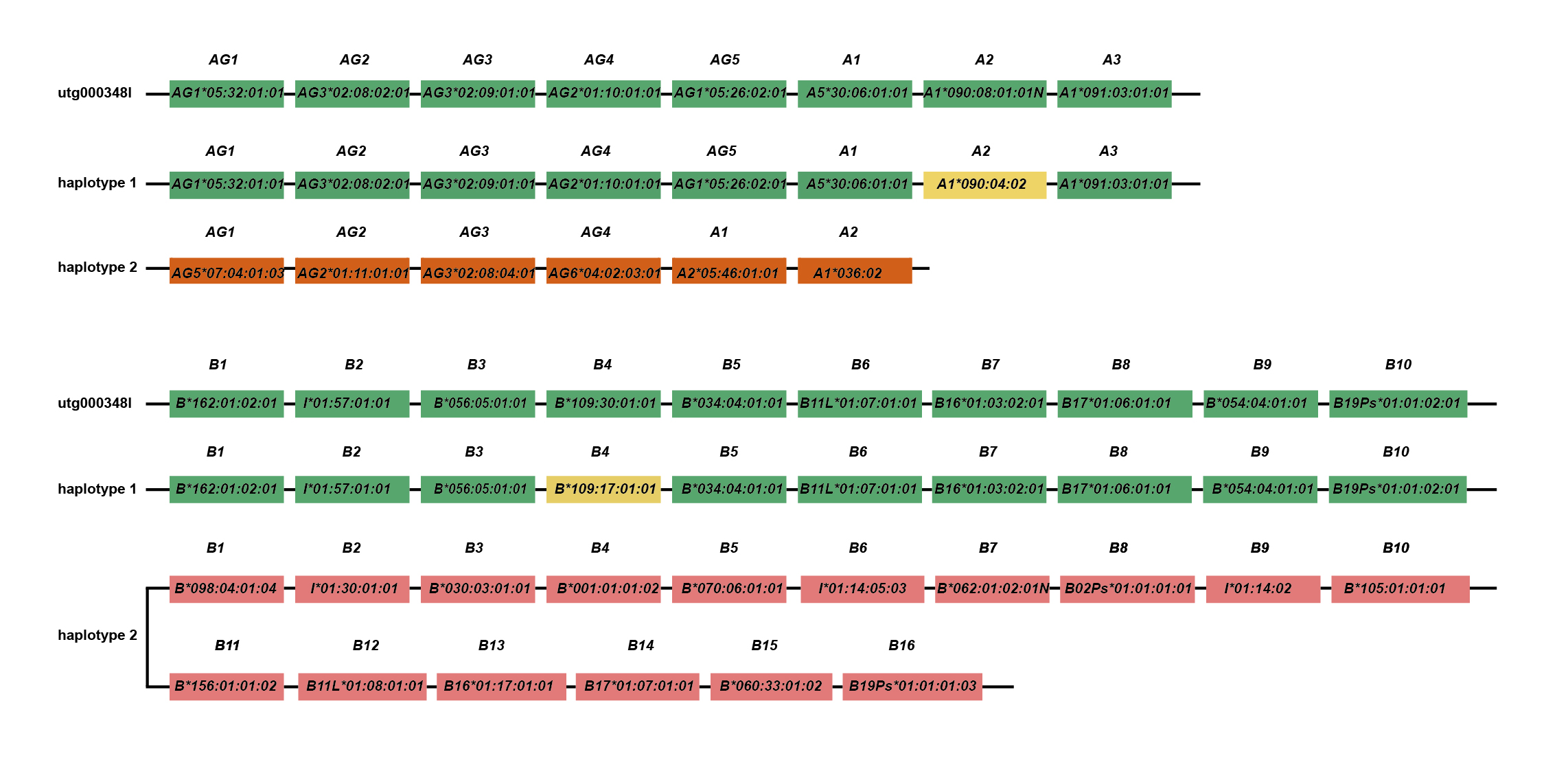
**

**Fig. S7 Linear representation of the cynomolgus macaque *MHC* genes in contig utg000348l and the two phased haplotypes.**

These *MHC* sequences in haplotype 1 and 2 were compared with the alleles in Immuno Polymorphism Database (IPD) to find the exons and introns of each allele. The novel sequences were received official designations. Distances between genes are not scaled.


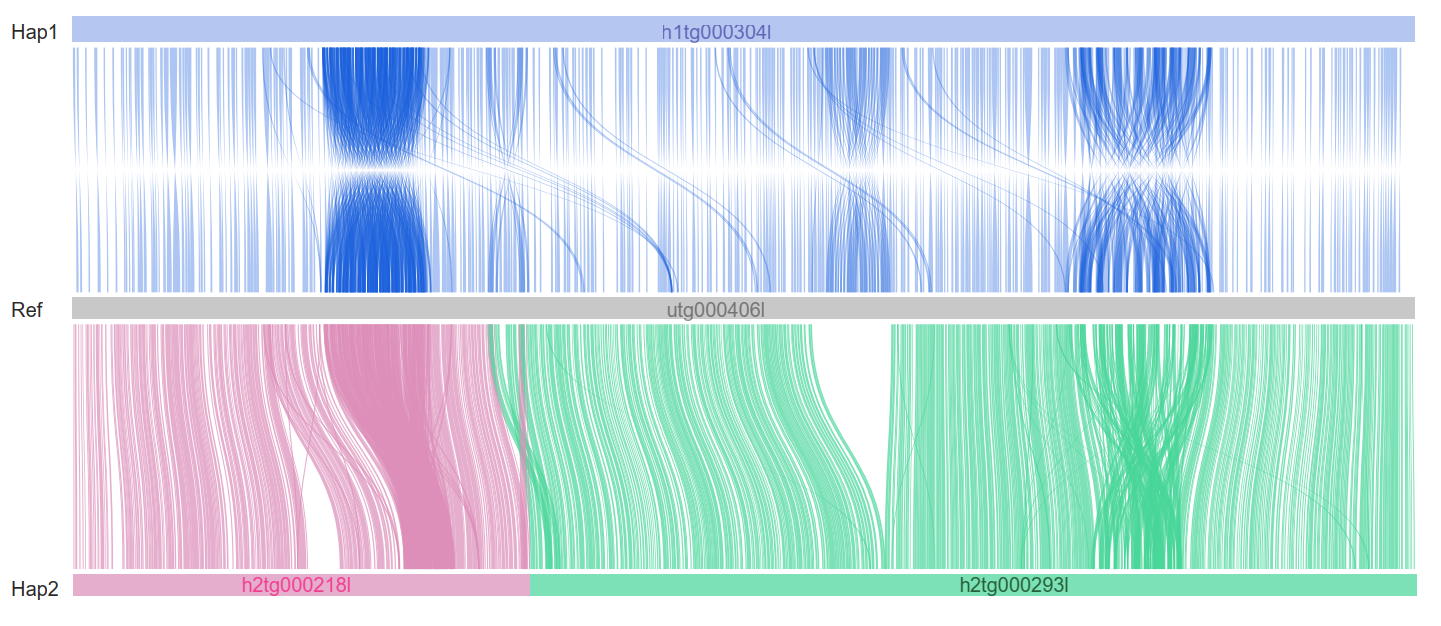


**Fig. S8 Collinearity analysis of KIR contig utg000406l and candidate KIR contigs in haplotype 1 and 2.** The KIR contig utg000406l was aligned to the assembled haplotype 1 and 2 using BLAST (v2.2.26). As a result, we obtained one candidate KIR contig and two candidate KIR contigs in haplotype 1 and 2, respectively. In haplotype 1, one contig, hltg000304l (blue), displayed collinearity with KIR contig utg000406l (gray). Some high-repetition areas have multiple comparisons, which are shown in the areas with dense lines plot. In haplotype 2, two contigs showed collinearity with the KIR contig utg000406l. They are as follows: h2tg000218l (only 330 kb displayed here; pink) and h2tg000293l (green). This figure shows only identity>0.95 and block>500 bp.


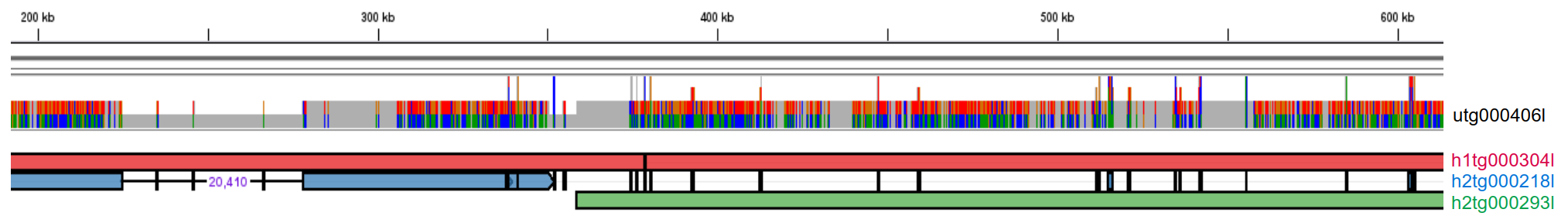


**Fig. S9 Sequence alignment of KIR contig utg000406l with candidate KIR contigs in haplotype 1 and 2**. The KIR contig utg000406l was aligned with three candidate KIR contigs (hltg000304l, h2tg000218l, and h2tg000293l) in haplotype 1 and 2 using Minimap2 (v2.24; r1122). Contig hltg000304l (red) in haplotype 1 is nearly identical to KIR contig utg000406l. In haplotype 2, h2tg000218l (blue) and h2tg000239l (green) have no significant overlap. There are clear insertions and deletions in the two contigs (h2tg000218l and h2tg000293l). Black bold lines indicate deletions (deletions>500 bp are shown). Lines in red, green, and blue on contig utg000406l indicate base mutations.


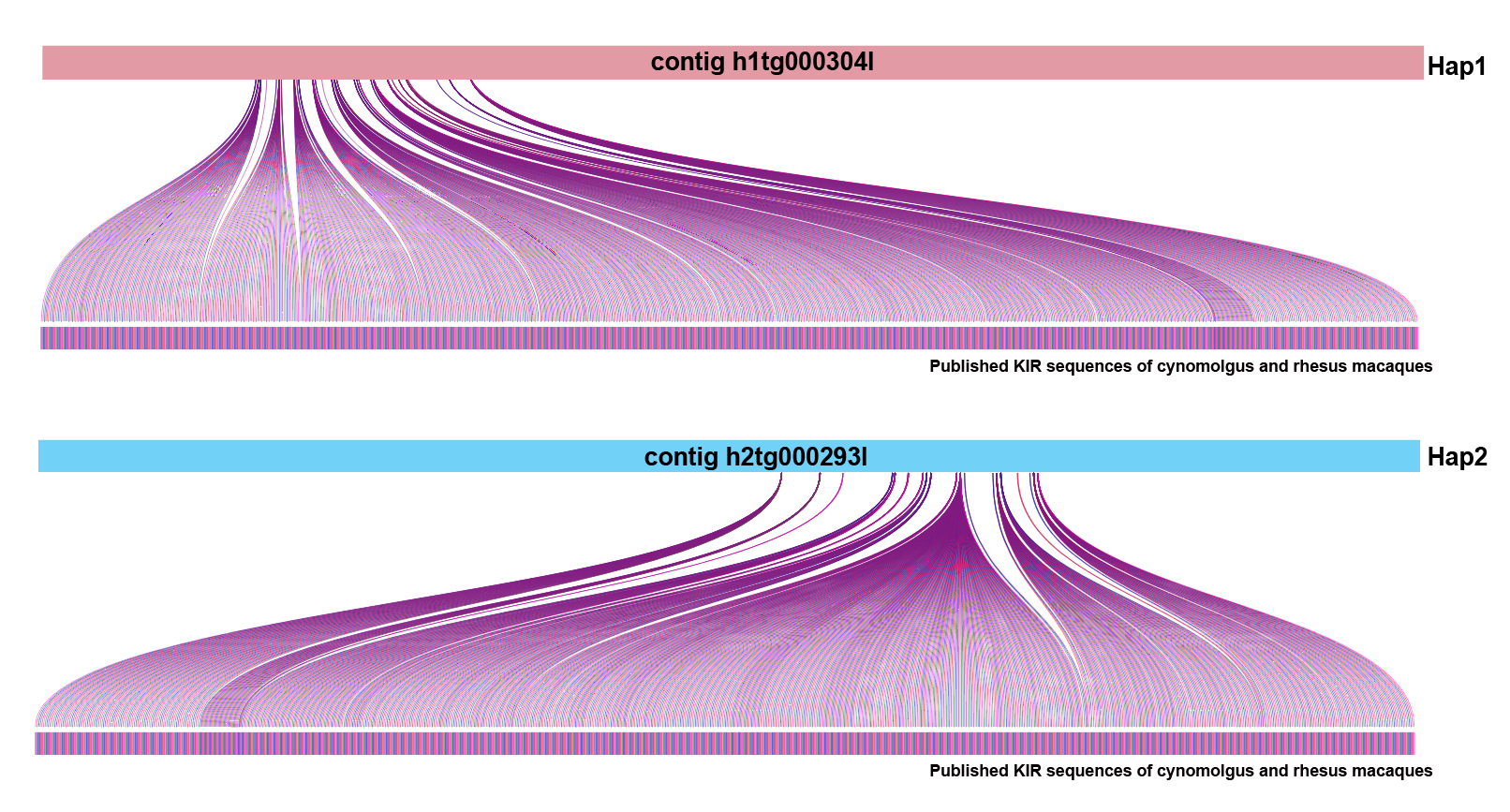


**Fig. S10 Collinearity between cynomolgus macaque candidate KIR contigs and published *KIR* sequences.** The *KIR* CDS sequences of cynomolgus and rhesus macaques downloaded from the IPD (purple) were collinearly compared with the three candidate KIR contigs hltg000304l, h2tg000293l and h2tg000218l using BLAST (v2.2.26). Contigs hltg000304l (pink) and h2tg000293l (blue) displayed collinearity with the *KIR* CDS sequences of cynomolgus and rhesus macaques downloaded from the IPD (purple). The comparison results showed that there was no *KIR* gene on h2tg000218l.


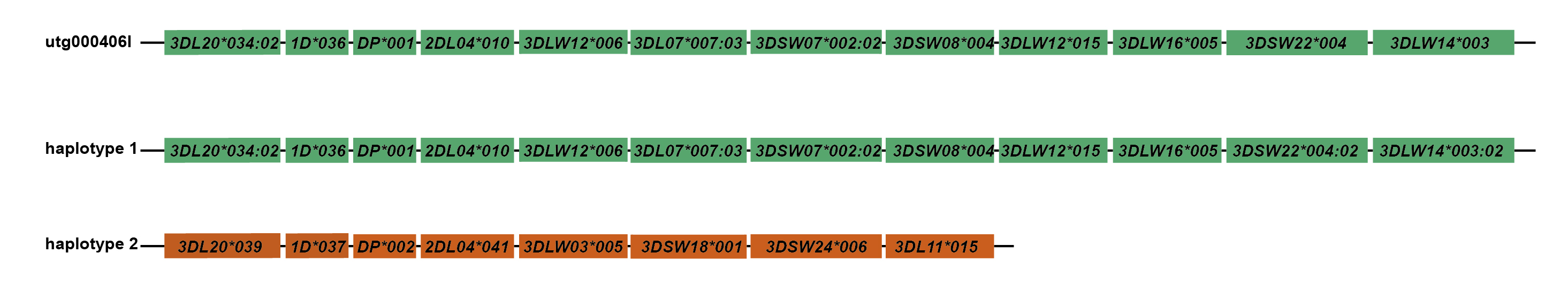


**Fig. S11 Linear representation of the cynomolgus macaque *KIR* genes in contig utg000406l and the two phased haplotypes.** These *KIR* sequences in haplotype 1 and 2 were compared with the alleles in Immuno Polymorphism Database (IPD) to find the exons and introns of each allele. The novel sequences were received official designations. Distances between genes are not scaled.

**Table S1** **Locus-specific primers for the ten different *Mafa-B* loci**

|  | **Primers** | | **Sequences (5’-3’)** | **Location** | **Tm**  **(℃)** | **Extension time (s)** | **Product**  **(bp)** |
| --- | --- | --- | --- | --- | --- | --- | --- |
| *B1* | *F1* | ACGCAGTTTGTGCGGTTCGACAGCA | | CDS2 | 58 | 47 | 847 |
|  | *R1* | AGCTCTTCCTCCTCCACATCACAGCG | | CDS5 |  |  |  |
| *B2* | *F2* | ATGCGGGTTATGGCGCCCGGA | | CDS1 | 64 | 25 | 498 |
|  | *R2* | GTTCTGAGCCGCCTCACCCGCA | | CDS3 |  |  |  |
| *B3* | *F3* | TCGCACTCCATGAGGTATTTCAGCAA | | CDS2 | 62 | 50 | 850 |
|  | *R3* | CGATGGGGATGGTGGACTGGGT | | CDS5 |  |  |  |
| *B4* | *F4* | GCTCGCACTCCTTGAGGTATTTCT | | CDS2 | 58 | 34 | 616 |
|  | *R4* | GCACCTCAGGGTGGCCTCATGGTT | | CDS4 |  |  |  |
| *B5* | *F5* | CCGTGTCCCGGCCCGGCCGGC | | CDS2 | 60 | 35 | 663 |
|  | *R5* | CACAAGTTCGGTGTCCTGAGTTTGA | | CDS4 |  |  |  |
| *B6* | *F6* | CCCACTCCATGAGGTATTTCC | | CDS2 | 60 | 35 | 673 |
|  | *R6* | TGAGTTTGCTCCTCCCCATCT | | CDS4 |  |  |  |
| *B7* | *F7* | ATGATGCCCGGAACCGTCCT | | CDS1 | 60 | 40 | 665 |
|  | *R7* | CAGGGTGGCCTCATGGTCAGAT | | CDS4 |  |  |  |
| *B8* | *F8* | CCTGGCCCTGACCGAGACCTGGGT | | CDS1 | 58 | 35 | 616 |
|  | *R8* | ATGGTCAGAGACGGGGTGGTGGGC | | CDS4 |  |  |  |
| *B9* | *F9* | GCGAACCCTCCTCCTGCTGCTCTCA | | CDS1 | 62 | 30 | 592 |
|  | *R9* | CAGCGTCTCCTTCCCGTTCTCCAT | | CDS3 |  |  |  |
| *B10* | *F10* | GCACCTCAGTGTCCCGGCCT | | CDS2 | 58 | 27 | 508 |
|  | *R10* | TGCAGCGTCTCCTTCCTGTTCTCT | | CDS3 |  |  |  |

“F” refers to forward primers, and "R" refers to reverse primers. “Tm” refers to annealing temperature.

**Table S2 Statistics of gene structure annotations in the assembled cynomolgus macaque genome**

|  | Gene set | Gene number | Gene length（bp） | | CDS number | Intron length（bp） | Exon length（bp） | Exon per gene |
| --- | --- | --- | --- | --- | --- | --- | --- | --- |
| Homolog | Homo_sapiens | 22,472 | | 45,097.48 | 1,633.71 | 5,462.87 | 182.41 | 8.96 |
|  | Macaca_fascicularis | 26,096 | | 40,988.78 | 1,576.80 | 5,193.50 | 183.59 | 8.59 |
|  | Monodelphis_domestica | 23,492 | | 51,308.56 | 1,453.95 | 7,686.00 | 194.21 | 7.49 |
|  | Mus_musculus | 22,612 | | 45,970.47 | 1,579.46 | 5,918.21 | 185.80 | 8.50 |
|  | Otolemur_garnettii | 25,285 | | 34,614.79 | 1,500.05 | 4,606.76 | 183.19 | 8.19 |
|  | Pan_troglodytes | 30,887 | | 37,933.39 | 1,438.38 | 5,247.19 | 180.81 | 7.96 |
| *De novo* | Augustus | 32,444 | | 26,115.37 | 1,194.70 | 4,678.97 | 188.85 | 6.33 |
|  | Genscan | 69,601 | | 33,916.22 | 1,149.04 | 5,756.33 | 171.69 | 6.69 |
| Transcript | Pasa+Transdecoder | 19,891 | | 28,760.12 | 1,010.42 | 4,789.03 | 148.71 | 6.79 |
| EVM |  | 31,606 | | 38,417.85 | 1,458.07 | 5,251.25 | 181.39 | 8.04 |

Three approaches were employed in gene prediction: homolog annotation, *de novo* prediction, and transcript annotation. EVM software was used to integrate the above three evidence sets, and to filter out genes based on the following conditions: a gene has only one kind of evidence supported by de novo prediction; the CDS length is short (<=150bp); and the overlap length ratio with TE is less than 0.2.

**Table S3 Statistics of functional genes** **in the assembled cynomolgus macaque genome**

| Database | Numbers | Percent |
| --- | --- | --- |
| Total | 31,606 | 100% |
| Swissprot | 23,594 | 74.65% |
| KEGG | 18,540 | 58.66% |
| TrEMBL | 20,640 | 65.30% |
| Interpro | 18,580 | 58.79% |
| Overall | 25,730 | 81.41% |

Functional annotations were performed using known protein libraries such as Swissprot, KEGG, TrEMBL, and Interpro.

**Table S4 BUSCO evaluation of the assembled cynomolgus macaque genome**

| Type | Percentage |
| --- | --- |
| Complete BUSCOs | 91.6% (2,370) |
| Complete and single-copy BUSCOs | 79.4% (2,054) |
| Complete and duplicated BUSCOs | 12.2% (316) |
| Fragmented BUSCOs | 4.4% (114) |
| Missing BUSCOs | 4.0% (102) |
| Total | 100% (2,586) |

Vertebrata_odb9 was selected as the evaluation database.

**Table S5** **Statistics of non-coding RNA genes in the assembled cynomolgus macaque genome**

| Type |  | Copy  (w) | Average length  (bp) | Total length（bp） | | % of genome |
| --- | --- | --- | --- | --- | --- | --- |
| miRNA |  | 1965 | 95.53 | 187,719 | 0.0051 | |
| tRNA |  | 705 | 74.90 | 52,801 | 0.0014 | |
| rRNA | rRNA | 1147 | 162.96 | 186,911 | 0.0051 | |
|  | 18S | 35 | 955.37 | 33,438 | 0.0009 | |
|  | 28S | 323 | 287.15 | 92,749 | 0.0025 | |
|  | 5.8S | 27 | 137.52 | 3,713 | 0.0001 | |
|  | 5S | 762 | 74.82 | 57,011 | 0.0016 | |
| snRNA | snRNA | 832 | 112.29 | 93,422 | 0.0026 | |
|  | CD-box | 194 | 98.79 | 19,165 | 0.0005 | |
|  | HACA-box | 99 | 132.65 | 13,132 | 0.0004 | |
|  | splicing | 474 | 118.49 | 56,162 | 0.0015 | |

Non-coding RNAs: miRNA (MicroRNA), tRNA (Transfer RNA), rRNA (ribosomal RNA), snRNA (small nuclear RNA).

Copy(w): copy number.

% of genome: proportion of the genome.

**Table S6** **Statistics of repeats in the assembled cynomolgus macaque genome**

| Type | Repeat Size（bp） | % of genome |
| --- | --- | --- |
| Tandem Repeat Finder | 86271994 | 2.36 |
| Repeatmasker | 1074965957 | 29.44 |
| Proteinmask | 344948794 | 9.44 |
| *De novo* | 1623390184 | 44.46 |
| Total | 1797430244 | 49.23 |

**Table S7 Transposable elements (TEs) content in the assembled cynomolgus macaque genome**

|  | Repbase TEs | Protein TEs | *De novo* TEs | Combined TEs |
| --- | --- | --- | --- | --- |
| Type | Length（bp） | Length（bp） | Length（bp） | Length（bp） |
| DNA | 48,563,422 | 7,290,899 | 23,728,600 | 65,892,709 |
| LINE | 493,803,800 | 294,768,425 | 794,664,790 | 931,652,606 |
| SINE | 361,359,567 | 0 | 424,611,122 | 590,006,356 |
| LTR | 173,644,721 | 42,922,217 | 293,548,021 | 416,809,359 |
| Other | 67 | 0 | 0 | 67 |
| Unknown | 0 | 0 | 3,340,129 | 3,340,129 |
| Total | 1,074,965,957 | 344,948,794 | 1,319,770,193 | 1,476,522,810 |

Transposable elements (TEs) of DNA, LTR (long terminal repeat), LINE (Long interspersed nuclear elements) and SINE (short interspersed nuclear elements) were counted. Repbase TEs: the result of RepeatMasker based on Repbase; Protein TEs: the result of RepeatProteinMask based on Repbase; *De novo*: repeats found *de novo* using Reaptmodeler; Combined: the results combining Repbase TEs and Protein TEs.

**Table S8 Statistics of phased haplotypes of cynomolgus macaque genome**

|  | Haplotype 1 | Haplotype 2 |
| --- | --- | --- |
| Total number （#） | 4,191 | 2,987 |
| Total length （bp） | 3,129,681,728 | 2,991,740,918 |
| Gap（bp） | 0 | 0 |
| Average length （bp） | 746,762.52 | 1,001,587.18 |
| N50 length （bp） | 16,911,907 | 15,048,870 |
| N90 length （bp） | 2,523,267 | 2,351,917 |
| Maximum length （bp） | 120,114,351 | 120,486,410 |
| Minimum length （bp） | 10,597 | 10,870 |
| GC content | 41.05% | 41.14% |

Two independent haplotypes were assembled by processing HiFi reads with hifiasm ( v0.12; -r1-x0.9-y0.2).
